# Earthquake segmentation in northern Chile correlates with curved plate geometry

**DOI:** 10.1038/s41598-019-40282-6

**Published:** 2019-03-13

**Authors:** Mahesh N. Shrivastava, Gabriel González, Marcos Moreno, Hugo Soto, Bernd Schurr, Pablo Salazar, Juan Carlos Báez

**Affiliations:** 1 National Research Center for Integrated Natural Disaster Management, Santiago, Chile; 2 Departamento de Ciencias Geológicas, Universidad Católica del Norte, Antofagasta, Chile; 3 GFZ Helmholtz Centre Potsdam, German Research Centre for Geosciences, Potsdam, Germany; 4 Centro Sismológico Nacional, Universidad de Chile, Santiago, Chile; 5 Departamento de Geofísica, Universidad de Concepción, Concepción, Chile

## Abstract

We performed an integrated analysis of the coseismic slip, afterslip and aftershock activity of the 2014 M_w_ 8.1 Pisagua earthquake. This earthquake seems to be spatially located between two major historical earthquakes, the 1868 M_w_ 8.8 earthquake in southern Peru and the 1877 M_w_ 8.5 earthquake in northern Chile. Continuous GPS data were used to model the coseismic slip of the mainshock and the largest aftershock (M_w_ 7.6). The afterslip was modeled for 273 days (end of year 2014) after the largest aftershock, revealing two patches of afterslip: a southern patch between the mainshock and the largest aftershock and a patch to the north of the mainshock. Observations from the seismic network indicate that aftershocks were concentrated near the southern patch. Conversely, the northern patch contained hardly any aftershocks, indicating a dominant aseismic slip. The Pisagua earthquake occurred within a prominent, curved section of the Andean subduction zone. This section may have acted as a barrier for the largest historical earthquakes and as an isolated segment during the Pisagua earthquake.

## Introduction

Theoretical models and earthquake observations reveal that subduction earthquake ruptures often stop their lateral propagation at specific places, which are called barriers^1^. Geodetic and seismic observations of recent large-magnitude earthquakes in subduction zones show that barriers are usually characterized by fault segments with low degrees of locking^2–4^, where the accumulated stress is low^5^. Barriers may also be regions that preferentially exhibit aseismic afterslip due to the presumed prevalence of velocity-strengthening friction at the fault surface^2,6^. With the improvement of earthquake observations, the quasi-stationary behavior of fault segmentation models has become obsolete. Earthquake segments rupturing together during large earthquakes (M_w_ > 8.5) have confirmed this concept and revealed the role of stress transfer from one fault segment to another^7^. In this manner, barriers can be considered as transient fault elements that can be partially or completely crossed by larger (M_w_ > 8.5) earthquakes. Although there are indications about the physical processes controlling the occurrence of barriers, it is still unclear under what circumstances the gaps between ruptures act as barriers to rupture propagation or alternatively act as nucleation points or even as a bridge to rupture neighboring asperities. It has been proposed that the occurrence of barriers may be controlled by several factors, including large-scale lithological heterogeneities^8^, the nonplanarity of fault surfaces^9^, or fluid localization^10^.

**Figure 1:**
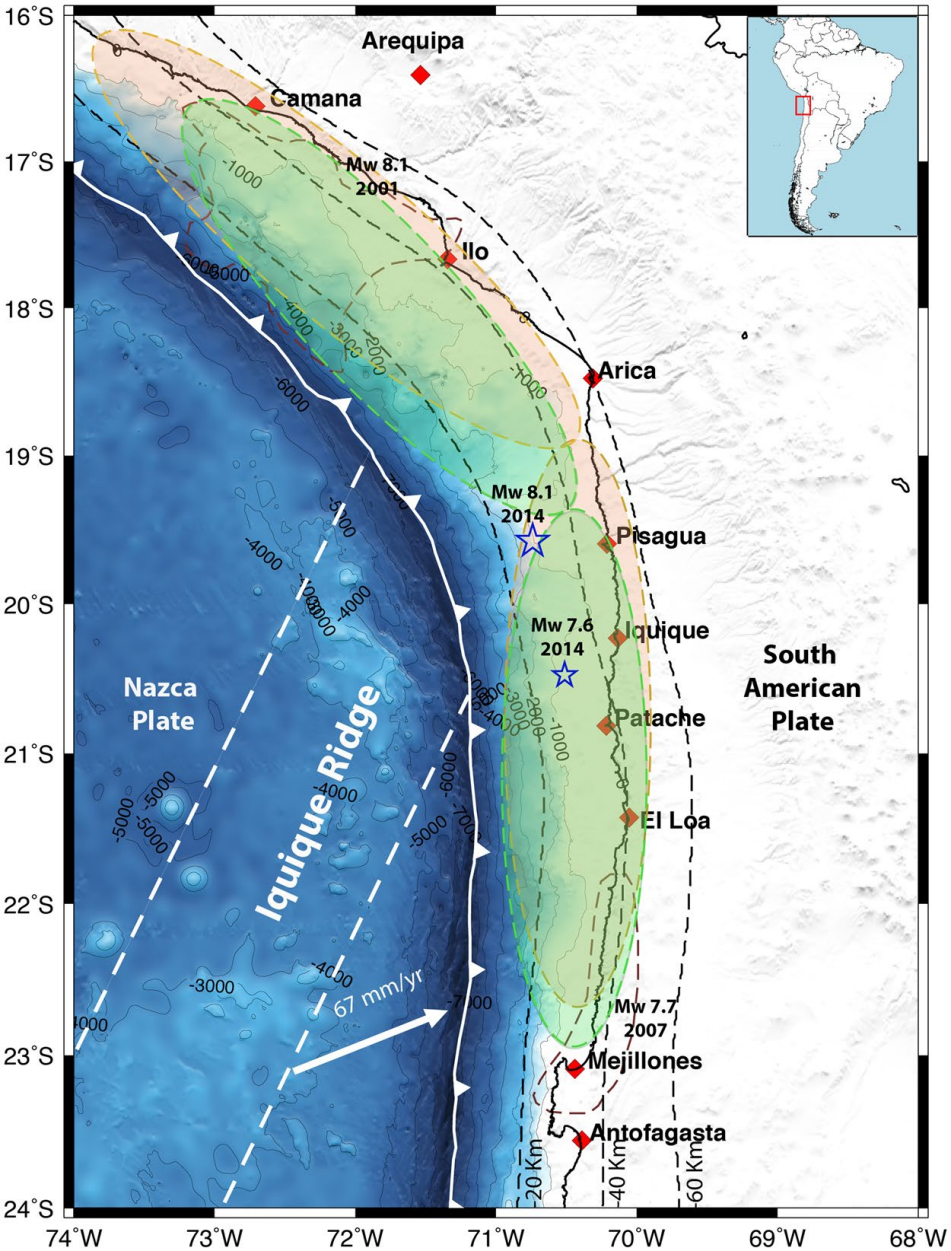
Seismotectonic map of the northern Chile and southern Peru subduction zone. The white line with triangles shows the trench that defines the boundary between the Nazca and South American plates. The orange and green ellipses indicate the alternative rupture area distribution of the two great historical subduction earthquakes in southern Peru (1868 earthquake) and northern Chile (1877 earthquake), according to intensity analyses performed by^12,49^, respectively. The extents of these two great earthquakes were identified by previous authors^12,49^ on the coast; thus, we represented the raptures in the off-coast area as a colored shaded region because these earthquakes generated significant tsunamis. The brown segmented lines indicate the rupture regions of the 2007 Tocopilla earthquake^52^ and the Arequipa earthquake^53^. The mainshock epicenter of the 2014 Pisagua M_w_ 8.1 earthquake and the epicenter of the largest aftershock (M_w_ 7.6) are shown with blue stars. This figure was generated using the Generic Mapping Tools^54^ version 4.5.8 (http://gmt.soest.hawaii.edu).

In this contribution, we focus on earthquake segmentation in a section of the Andean subduction zone where the shallow part (0–40 km depth) of the plate margin is characterized by a significant curved plan view extending through southern Peru and northern Chile (Fig. 1). In this tectonic setting, a long-standing seismic gap was partially broken on April 1^st^, 2014, by the M_w_ 8.1 Pisagua earthquake^4,11^. This area had remained unruptured since the two major historical earthquakes that occurred in southern Peru and northern Chile, the 1868 M_w_ 8.8 and the 1877 M_w_ > 8.5 (Fig. 1) earthquakes, respectively^12^. Several authors have documented the accumulated moment deficit in this seismic gap, suggesting that this area could host a M_w_ 8.9 earthquake similar in magnitude to the most recent great historical earthquakes^11,13,14^. However, the 2014 Pisagua earthquake was smaller than predicted; consequently, a high slip deficit still remains in the northern Chile region with the potential ability to generate a M_w_ 8.8 earthquake^4^. Teleseismic and geodetic inversion models^4,15,16^ reveal that the coseismic slip pattern of the mainshock was characterized by a single slip patch located offshore of Pisagua. The most energetic aftershock, a M_w_ 7.6, occurred on April 3rd, 2014, at the southern boundary of the main rupture, ~27 hours after the mainshock. A 2-year postseismic model^17^ suggests that afterslip quickly dissipated and that relocking restarted in the region affected by the Pisagua earthquake. One question that remains unanswered is why this seismic gap did not completely break during the Pisagua event. In this work, we try to answer this question by studying in detail the coseismic and the first 9 months of postseismic deformation after this earthquake. We used continuous time-series GPS data to model the coseismic slip of the mainshock and the largest aftershock and to evaluate the afterslip process. Furthermore, seismic data provided by a high-resolution network of seismic stations were used to characterize aftershock activity for 273 days after the largest aftershock. We propose that margin curvature played a major role in the historical earthquake segmentation. Although the exact location of the two large historical earthquakes remains still a matter of debate, the Pisagua earthquake seems to be located at the junction zone of the two large historical earthquakes. Therefore, it appears that the most curved portion of the margin arrested the propagation of the two major historical earthquakes by acting as a barrier and also acted as a segment during the Pisagua earthquake. Therefore, we suggest that the curved portion of the Andean margin can behave dually as a barrier and as a single rupture segment. Many subduction zones on Earth show similar major, along-strike curvature, e.g., the northern Cascadia subduction zone and along the northeastern Japan trench, south of Hokkaido. Therefore, the conclusions obtained in this work can be applied to understanding earthquake segmentation in subduction zones with complex curvature parallel to the trench.

## Methodology

### GPS Data Processing

For coseismic displacements, we computed the daily positions of 19 cGPS stations in two steps using GAMIT/GLOBK software (Massachusetts Institute of Technology, Cambridge, USA)^18,19^. These stations are operated by different research groups including IPOC, Caltech, CSN Chile and ISTerre^11,20,21^. The details, names, distribution and processing of cGPS data are presented in Tables S1 and S2 and in the Supplementary Information. In the first step, loose daily GAMIT results were obtained, which accounted for error contributions due to signal delay by the atmosphere, orbital accuracy, antenna phase center variations, signal multipath and satellite as well as receiver clock errors. Ambiguity-free and ambiguity-fixed solutions were executed with ionosphere-free linear combinations to account for carrier phase ambiguity and signal delay due to the ionosphere. We utilized IGS tables to correct the positions of the phase centers of antennas and estimated the tropospheric vertical delay parameter per station every 3 hrs. Precise orbits and earth rotation parameters were obtained from the International GNSS Service (IGS) for Geodynamics^22^. The GPS site positions were estimated daily and independently using a weighted least square technique. In the second step, loosely constrained daily solutions from global tracking IGS sites were combined with everyday solutions obtained from GAMIT, resulting in loosely constrained locations for the entire survey. These combined solutions were passed through a Kalman filter with GLOBK using a local stabilization method^16^ to estimate network-adjusted site coordinates. The horizontal (East and North) and vertical (Up) components, relative to the position vectors, are precise within 2–3 and 4–5 mm, respectively. We utilized the GPS sites KOUR in French Guyana, BRAZ, BRFT and CHPI in Brazil, and RIO2 in Patagonia and GLPS on the Nazca plate, which were not displaced from the Maule earthquake in 2010^23^. The genuine GPS sites GLPS, KOUR, BRAZ and RIO2 were utilized for constraining our solutions. The International Terrestrial Reference Frame (ITRF) 2008 was executed through GLORG using locally produced h-files. This stabilization strategy defines a reference frame by reducing, using a least squares rationality, the departure from the a priori data determined in the ITRF2008^24^. The GPS time series are shown in Fig. S9.

### Inversion Modeling

**Figure 2:**
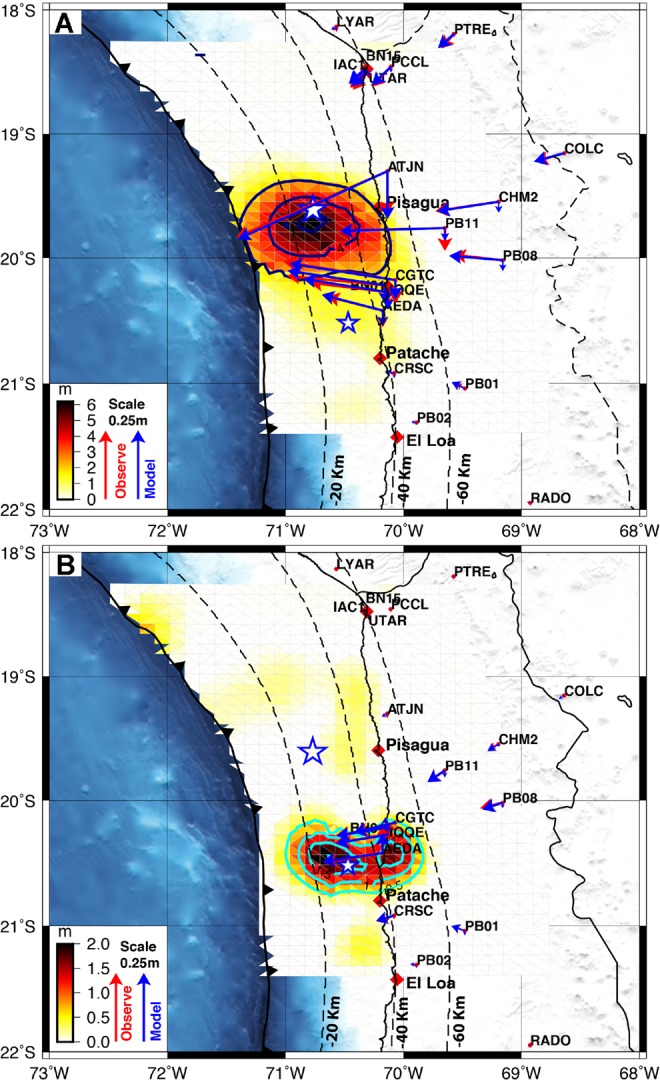
(**A**) Coseismic slip inverted from cGPS data from the Pisagua M_w_ 8.1 mainshock on April 1^st^, 2014. The red and blue arrows show the observed and modeled GPS displacements, respectively. Slab megathrust contours (every 20 km of depth) are taken from slab model 1.0^23^. The dark blue closed line shows the 2 m contours of coseismic slip of the mainshock. (**B**) Coseismic slip inverted from cGPS displacements from the M_w_ 7.6 aftershock on April 3^rd^, 2014. The cyan closed lines indicate 0.50-m contours of coseismic slip of the largest aftershock. The epicenters of the 2014 Pisagua mainshock (M_w_ 8.1) and the largest aftershock (M_w_ 7.6) are shown with blue stars. This figure was generated using the Generic Mapping Tools^54^ version 4.5.8 (http://gmt.soest.hawaii.edu).

For coseismic slip and afterslip modeling, we inverted the cGPS (E-N-U) position components of the time series by using the principal component analysis method (PCAIM)^2,25^. The cGPS sites recorded large horizontal displacement towards the trench associated with both the mainshock on April 1^st^, 2014, and the largest aftershock on April 3^rd^, 2014 (Fig. 2A,B and Tables S1 and S2; see the methods section for raw GPS data processing). Using the inverted cGPS time series, we modeled the coseismic slip of the mainshock and the largest aftershock separately (shown in Fig. 2A,B).

Afterslip was modeled geodetically for a period of 273 days after the largest aftershock. We assume in our modeling strategy that in this period, postseismic relaxation is dominated by an elastic behavior. We were not able to reproduce the afterslip immediately after the mainshock because the GPS signals were unstable due to the largest aftershocks. Therefore, our afterslip model starts ~30 hours after the mainshock and covers the subsequent 273 days. For modeling the afterslip distribution, we used one solution for one-day GPS data, from April 3^rd^, 2014 (after the largest M_w_ 7.6 aftershock), for 273 days (Table S3 presents the cumulative displacement). We used the Slab1.0 model^26^ of subduction zone geometry to represent the megathrust in the models. The seismogenic zone in this region ends approximately at a depth of 60 km^27^. Accordingly, we stretched the megathrust fault from 21.4°S to 18.2°S along-strike and from the trench axis down to a depth of 100 km. The slab 1507 triangular elements with an area ~67 km^2^ is embedded in a homogeneous half-space with a shear modulus of 35 GPa and Poisson’s ratio of 0.25. The slip on the triangular elements was regularized using a Laplacian operator to smooth the final slip distribution on the fault, rooted in the L-curve^28,29^. We implemented the boundary conditions of the slab fault along the trench to fix the slip at zero, while other boundaries of the fault were not fixed. A series of checkerboard tests were performed to estimate the resolution of the inversion models (Figs. S1 and S2). The coseismic displacements of the mainshock, the largest aftershock and the cumulative displacement are provided in Tables S1–S3 in the Supplementary Information.

### Estimation of Coulomb Stress Changes

The Coulomb stress changes (CSC) introduced by the mainshock were computed using Coulomb 3.3^30,31^, assuming uniform, isotropic elastic half-space (i.e., ignoring the effects caused by the layered earth or inhomogeneous 3D structure) to simulate the Earth’s media. The estimation of Coulomb stress is based on the faulting parameters of the mainshock (strike/dip/rake), the fault slip and the apparent coefficient of friction in the crust^32^:

1 $${\rm{\Delta }}{\sigma }_{f}={\rm{\Delta }}{\tau }_{s}-\mu ({\rm{\Delta }}{\sigma }_{n}-{\rm{\Delta }}P)$$

where the μ is the coefficient of friction, Δτ_s_ is the change in shear stress, Δσ_n_ is the change in normal stress and ΔP is the pore pressure change (not considered in this analysis). The Coulomb stress change is computed using the modeled distribution of coseismic slip of the mainshock and largest aftershock at the plate interface, shown in Fig. S4.

### Aftershock Analysis

In our postseismic deformation analysis, we utilized aftershock data from published seismic catalogue^33^. This catalogue contains 4706 aftershocks with local magnitude of at least (Ml) < 1.4 and complete magnitudes above ~2.8 (Fig. S4B,D). These data have been collected by permanent seismic network of the IPOC and CSN. The monthly distributions of seismicity from south to north are presented in the Supplementary Information (Fig. S6). To understand how the evolution of afterslip relates with aftershock activity, we fitted the aftershocks activity with a modified Omori’s law (Fig. S6A) and estimated the seismic moment released monthly (Fig. S7). To better determine the aftershocks distribution, we used an additional data for aftershocks provided by a temporary seismic network installed shortly after the mainshock. We relocated the aftershocks that occurred within the two first months of the aftershock period. Aftershocks were relocated by searching for correlated signals across the temporary and permanent seismic network (Table S4) on a three-dimensional location grid accounting for predicted travel times. Arrival times and locations were then refined using an automatic, multistage procedure that applied dedicated phase-pickers and rigorous quality control^34^. As a final step, cross-correlation-based differential travel times were added, and all events were relocated using the double-difference relocation algorithm^35^, yielding 1857 aftershocks with local magnitude of at least (Ml) < 2.7. The relocated aftershocks are shown in the Supplementary Data (Fig. S4(C)).

## Results

### Coseismic Slip and Afterslip

The coseismic slip was concentrated in a single patch extending approximately 95 km along-strike and 130 km downdip, located to the south of the mainshock epicenter. The slip of the largest aftershock was distributed south of the mainshock rupture. The 6.24 m peak coseismic slip of the mainshock occurred at 19.7°S, −70.8°W at a depth of approximately 23 km (Fig. 2A). The checkerboard test suggests that our coseismic model is well resolved from a depth of 23 km downward to 60 km. Regions near the trench have a bad resolution; thus, these regions are not considered in further discussion. Our coseismic model resolves well the peak slip depth, being similar in magnitude and position that previous published models (GPS^15^, GPS-teleseismic^4^, teleseismic^36^ and tsunami^37^), joint inversion models^16^ using high-rate GPS (HrGPS) and strong motion places the maximal slip downward to 25 km depth (Fig. S3). One important aspect is that all of the models indicate that the main coseismic slip was located in a range of depth between 30–50 km and that it did not propagate near the trench, with an updip rupture boundary at 18–20 km in depth. The joint inversion models^16^ (HrGPS and strong motion) define a better resolution of the coseismic slip. In these models, the slip is more concentrated, and therefore, peak slip appears higher, reaching up to 10 m. It is consisting with a rupture dominated by one main asperity. Those models also suggest a secondary asperity located southeast of the mainshock epicenter. The moment release function^16,38^ indicates that one large asperity was the energetic one during the mainshock. All the models coincide in to define the position of this energetic asperity.

The rupture of the largest aftershock was placed southward of the main rupture and propagated beneath the coastal line. According to the checkerboard test (see Fig. S1), the coseismic slip of the largest aftershock is well resolved. The maximal slip during this aftershock was concentrated in two zones: one reaches 2.01 m of slip and located at 23 km depth, and the second one reaches 1.66 m of slip and located at 38 km depth, beneath the coastal line (Fig. 2B). A similar type of slip was obtained by previous works^38^. See Tables S1 and S2 in the Supplementary Information for more details about the coseismic displacements and the model predictions of the mainshock and the largest aftershock coseismic displacements.

**Figure 3:**
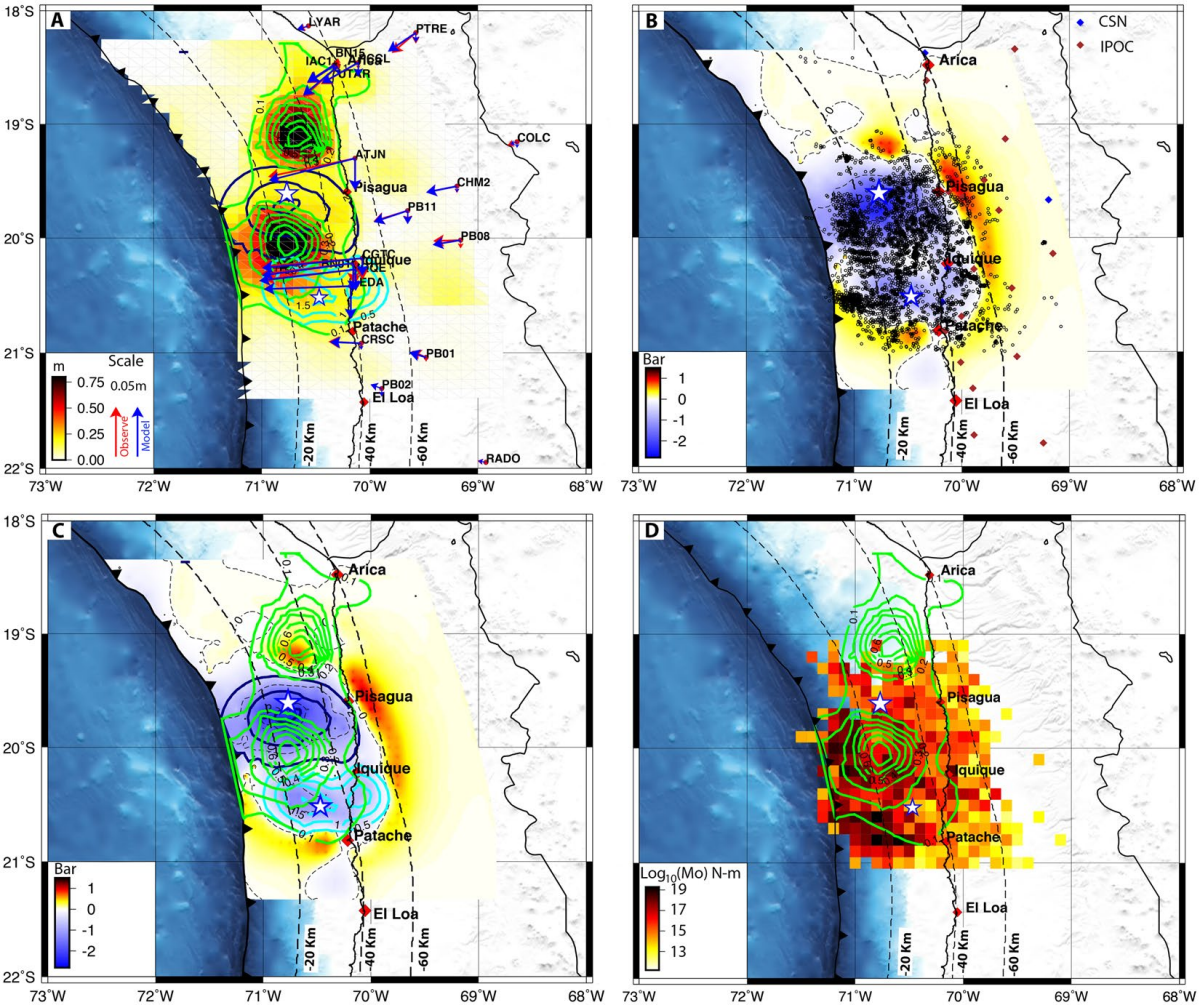
(**A**) Postseismic afterslip (0.10 m contour, green) inverted from 273 days of cGPS data. In those 273 days, most of the afterslip occurred north and south of the coseismic slip within two patches with a maximum of 76 cm of slip in the northern patch, occurring between depths of 30 and 38 km. The dark blue and cyan closed lines show the coseismic slip 2 m during the mainshock and 0.50 m the largest aftershock, respectively. (**B**) The combined Coulomb stress changes estimated from the coseismic slip of the mainshock and the largest aftershock along the megathrust. The aftershock distribution is plotted on the Coulomb stress change map. The seismic networks are depicted with colored diamonds. (**C**) The combined Coulomb stress changes estimated from the coseismic slip of the mainshock and the largest aftershock with variable rake angles along the megathrust. The green 0.10-m contour lines of afterslip are mapped on the Coulomb stress change map. The dark blue and cyan closed lines show the coseismic slip 1 m during the mainshock and 0.50 m the largest aftershock, respectively. (**D**) The seismic moment released distributed in cells (0.5 degree) from the aftershocks from April 3^rd^, 2014, to the end of the year 2014. The green 0.10-m contour lines of the afterslip are mapped on the seismic moment released map. This figure was generated using the Generic Mapping Tools^54^ version 4.5.8 (http://gmt.soest.hawaii.edu).

The afterslip from the inverted cGPS time series for the 273 days of postseismic relaxation exhibits temporal continuity decay, and the cumulative magnitude of afterslip increases with time. The afterslip model indicates that following the largest aftershock, afterslip mainly occurred in two separated patches, one located at the northern main rupture boundary and another at the southern rupture ends (Fig. 3A). The northern patch (patch A) is located at a depth of 38 km, whereas the southern patch (patch B) occurs at the boundary between the mainshock and the largest aftershock, at a depth of 20 km. Our resolution tests indicate that north patch is well resolved. The south patch is confined in depth between 10 to 35 km, half of this patch is well resolved by our model, and the peak slip of this patch is placed in the region where the checker board tests indicates a good resolving power of the inversion. The northern patch accumulated a maximum slip of 76 cm, whereas the southern patch had a maximum slip of 78 cm. The cumulative displacements of GPS during the afterslip period were at their maximum (10.27 cm) in the coastal area of Iquique, right eastward of the southern patch (Fig. 3A) at GPS site CGTC. In this part, the effects of the mainshock and the largest aftershock in the GPS signal are overlapping. The ATJN GPS site shows significant horizontal displacement of ~8.9 cm. This GPS site is located far from the largest aftershock rupture area; thus, this station captures mainly the short postseismic effect of the mainshock. In the north, GPS station displacements decreases around one-fourth of the maximum (Fig. 3A). The afterslip evolution has also been modeled monthly from April to December, as shown in Fig. S5A,B in the Supplementary Information, respectively. The mainshock slip yielded a geodetic seismic moment of M_o_ = 1.98 × 10^21^ N m, corresponding to a magnitude of M_w_ = 8.13, and the largest aftershock yielded 3.22 × 10^20^ N m, equivalent to a M_w_ 7.6 earthquake. The cumulative postseismic moment released as afterslip (during the 273-days following the largest aftershock) was 3.27 × 10^20^ Nm, which is approximately 16.5% of the mainshock and corresponds to a magnitude of M_w_ 7.61.

### Distribution of Aftershocks and Afterslip

Omori’s law indicates that aftershock activity decays after 9 months to the background seismicity^33^. To better understand the relationship between seismic and aseismic slip in the postseismic period, we calculated the seismic moment released from published seismic catalog^33^ (Fig. 3D). In the 273 days following the largest aftershock on April 3rd, there were 4706 aftershocks that released an equivalent seismic moment of 1.37 × 10^20^ N m (~ M_w_ 7.35). By comparing this amount with the cumulative seismic moment released as afterslip, we conclude that only ~41.8% of the postseismic afterslip occurred due to seismic processes and that the remaining 58.10% was released aseismically.

The CSC induced along the megathrust by the coseismic slip of the mainshock and the largest aftershock ranged from +0.46 to −2.76 bars, as shown in Fig. 3B. The lowest Coulomb stress changes were confined to the maximum slip regions of the mainshock and the largest aftershock. In the case of afterslip, models show that patch A occurred in the region of positive CSC. Patch B is localized in a region with less negative CSC, and we interpreted that this situation is given by the poorer resolution of afterslip model near the trench. The correlation of aftershocks and CSC shows that these events were not distributed entirely within areas of positive CSC (Fig. 3B,C). The location of some aftershocks in the areas of negative CSC is most likely combined effect of the poor resolution of the coseismic slip model near the trench and the promotion of aftershocks during progression of afterslip. Near patch A, where the seismic temporary and permanent network is more capable of detecting events, there were very few aftershocks, indicating that occurrences of the afterslip were dominantly aseismic. Conversely, aftershocks also occurred in patch B, but the contribution of aftershocks to the release of a seismic moment during the 273-day postseismic phase was ~69%. Our analysis indicates that patch A was dominated by an aseismic slip, suggesting a rate-strengthening frictional behavior, whereas patch B exhibited a seismic and aseismic slip, suggesting mixed rate-strengthening and rate-weakening frictional behavior. The cumulative displacements of GPS sites during the 273-day postseismic period shows that the coastal GPS sites IQQE, BN01, AEDA and CGTC near the city of Iquique exhibited the higher postseismic displacements, evidencing the occurrence of higher afterslip in this section of the megathrust. Towards the north, the cumulative displacement of GPS sites decreases due to a minor amount of afterslip.

To estimate the relaxation time of the afterslip, we followed the rate-strengthening frictional sliding law^39^. This law provides the evolution of an afterslip U(t), as shown in Eq. (2):

2 $$U(t)={V}_{pl}{t}_{r}\,{log}[1+(\frac{{V}^{+}}{{V}_{pl}{t}_{r}})t]$$

where V_pl_ is the plate velocity, t_r_ is relaxation time, V^+^ is the sliding velocity during the afterslip process, and t is the time since the mainshock. The evolution of the afterslip and the seismic moment of aftershocks calculated monthly is shown in the Supplementary Information, Figs. S5 and S7. By using Eq. (2), the best-fit model for each patch shows that the relaxation time of patch A was approximately 1.82 years and 1.78 years in patch B. The V^+^/V_pl_ ratio for patch A is 120; for patch B, 110. Thus, the sliding velocities at the plate interface during the afterslip are 2.86 × 10^−5^ cm/s for patch A and 2.28 × 10^−5^ cm/s for patch B (see Supplementary Information Fig. S8).

The comparison between the relaxation time of afterslip and aftershocks decay indicates that the afterslip velocity decelerates quickly in the first 20 days, and then it stabilizes. The aftershock activity also decreases drastically in the first 20 days after the mainshock, and then, the decay rate of aftershocks is decelerating drastically. These decay patterns suggest that afterslip and aftershock activity were coeval, particularly in areas surrounded the patch B, where the cumulative moment released was higher. In terms of the fracture process, these coeval patterns illuminate how transient stress is created by afterslip progression.

## Discussion

**Figure 4:**
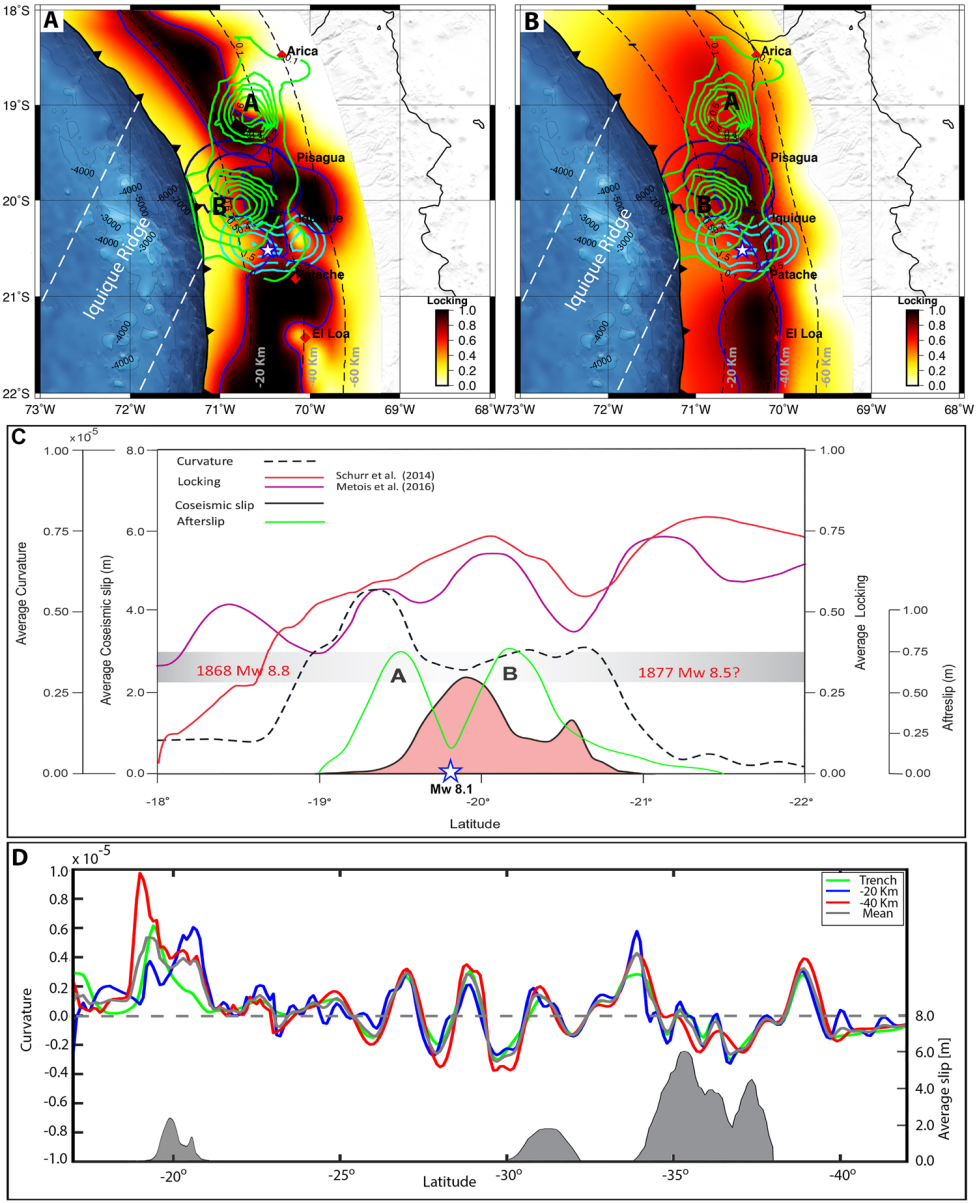
(**A**) Interseismic published coupling model^14^ derived from interseismic GPS data. The blue contour shows the 75% locking degree. (**B**) Interseismic coupling model^4^ derived from interseismic GPS data. The blue contour shows the 75% locking degree. The dark blue and cyan closed lines show the coseismic slip 2 m during the mainshock and 0.50 m the largest aftershock of Fig. 2A,B. (**C**) The correlation of published interseismic locking models, coseismic slip, afterslip and curvature from the present study. The light pink area shows the average coseismic slip, and the light blue and red lines show the locking models of^4,11^, respectively. The green line shows the afterslip distribution with scale. The black dotted line shows the along-strike mean curvature of the trench contours at slab depths of 20 km and 40 km. (**D**) Figure shows the curvature of the Nazca and South American plate boundary. The green line shows the curvature of the trench, the blue line shows the curvature of the slab contour at −20 km, and the red line shows the curvature of the -40-km slab contour. The thick gray line shows the mean curvature of trench and the 20- and 40-km slabs. This figure was generated using the Generic Mapping Tools^54^ version 4.5.8 (http://gmt.soest.hawaii.edu).

In general, afterslip is mostly observed downdip and updip of the rupture area of a subduction earthquake^2^. In early formulations, afterslip was assumed to be dominated by aseismic slip^6^. However, more recent studies using denser networks of seismic stations have demonstrated that modeled afterslip correlates with locations of aftershocks^40–42^, indicating that aseismic and seismic processes operate together. In the case of the Pisagua earthquake, we observed distinct behaviors in each patch of afterslip; patch B slipped due to a combination of seismic and aseismic processes, and most of the aftershocks surrounded this patch. The northern patch A slipped purely aseismically, and no aftershock activity was detected in this patch. More specifically, patch A most likely represents a section of the plate interface that was markedly less coupled preceding the Pisagua earthquake^4,11^. It is widely accepted that fluid flow in subduction zones drastically reduces the coupling degree in the updip and downdip ends of the seismogenic zone^10,43^, thus promoting slow slip processes. The location of patch A coincides well with the place where the margin attains its maximal curvature, it suggests that along-strike plate curvature plays a significant role in plate locking. In the case of the observed postseismic period, we suggest that the aseismic behavior of patch A may have been influenced by high pore fluid pressure. Existing locking models show that the area of patch A coincided with a region of reduced coupling (<40%, Fig. 4A–C). This section is most likely enriched in fluids released from metamorphic dehydration reactions occurring in the downdip part (40 km) of the seismogenic zone. An excess of fluid pressure can drastically reduce the frictional strength of the megathrust at this depth^44^.

According to published interplate locking models^4,11,14^, the mainshock breaks a highly locked zone of the interplate contact (Fig. 4), and the largest aftershock ruptures the boundary between the highly locked zone and an area characterized by lower locking at 20° 46′S. Particularly, the largest aftershock ruptured down dip unlock regions of the megathrust, this situation can be interpreted as a result of static CSC caused by the mainshock as it has been demonstrated by a previous study^45^.

The two afterslip patches end in less-locked regions enclosing the mainshock. A great part of the coseismic slip region also slipped during the afterslip period, particularly in areas of patch B. Fig. 4 summarizes our results; it shows, additionally, that the coseismic slip and afterslip regions were located along a portion of the plate boundary characterized by maximal curvature of the interplate contact (<40 km depth) along-strike. Along-trench variations in the average interplate locking illustrates that the boundaries of the coseismic slip ended in places where interplate locking was reduced, suggesting that interseismic locking exerted control over the earthquake propagation. The two patches of afterslip also coincided with these sections of lower degrees of locking. This result suggests that interplate margin geometry played some strong control on the locking. However, the most-curved part of the margin coincides with the subduction of the Iquique ridge^46–48^. Previous works have identified the region where the main rupture stops and where the largest aftershock was located as a region exhibiting several tectonic particularities^47,48^. In this region, a strong gravity gradient has been proposed^48^, which coincides with the position of the NE-SW-trending southern border of the Iquique ridge (Fig. 1). Furthermore, at 21°S, the interplate contact between the trench and the iso-depth contour of 50 km changes its geometry along the dip, from north to south^48^. North of 21°S, the interplate contact is flat from the trench to a depth of 50 km, whereas south of 21°S, the interplate contact sharply changes in dipping angle at 30 km in depth, being higher below 30 km. All of these changes can be ascribed to the subduction of the Iquique ridge, which affects the geometry of the plate interface. This ridge most likely also exerted a control in the rupture behavior of the Pisagua earthquake and in the distribution of afterslip patches. In fact, high-resolution bathymetric data show that the Iquique ridge contains several seamounts^47^, which could act as areas of reduced coupling contributing to coupling reduction and to aseismic process due to fluid infiltration. These areas can be preferred for hosting the aseismic process during postseismic relaxation.

We propose a general model in which the margin curvature of southern Peru and northern Chile and the Iquique ridge control earthquake segmentation at regional scale. Straight segments allow for hosting giant (M_w_ > 8.5) ruptures. The most-curved part could exhibit a mixed behavior, as barrier for these giant earthquakes and as a single segment for smaller earthquakes (M_w_ < 8.1). Following this idea, the historical 1868 M_w_ 8.8 earthquake and the 1877 M_w_ 8.5 earthquake broke the straight portion of the margin. In Fig. 1, we have reproduced the rupture extent of these earthquakes estimated using isoseismal data^12,49^. Although the exact distribution of the rupture of these earthquakes is still in debate, existing models^12,49^ agree that these earthquakes stopped their lateral propagation in the most-curved part of the margin, where the Pisagua earthquake was located. A previous contribution^11^ has described this region, where the Pisagua Earthquake struck as an earthquake segment, referred as the Camarones Segment. This segment is separated at 21°S from a southern segment, called the Loa Segment, by an area of reduced locking. According to this previous contribution, this segment hosted the 1877 earthquake. Therefore, we propose that the junction zone of these two historical earthquakes represents a seismic barrier for the two great historical earthquakes. In this context, these two large historical earthquakes may have ruptured fully locked and straight segments localized in southern Peru and northern Chile (Fig. 4). Presently, the most-curved part of the margin exhibits several particularities in terms of seismic/frictional processes; e.g., during the preseismic stage of the Pisagua event, it was dominated by repeating earthquakes^50^ and by aseismic pulses^15^. Previous studies^14,15^ identified that after the intraplate 2005 Tarapacá earthquake the eastward velocity at UAPE GPS site decreased by 20%, from 19.5 to 15.2 mm/year. The velocity reduction in this GPS site seems to reflect a reduction of convergence velocity controlled by slow slip events in this part of the margin^14,15^. It suggests that the areas of concentrated aseismic slip observed during the afterslip period were also able to concentrate aseismic slip at decadal scale (2005–2014). These observations strongly suggest that the curved section of the megathrust hosts significant temporal and spatial variations in terms of rate-state friction laws, behaving dually as a barrier during the two large historical ruptures and as a single segment during the Pisagua earthquake.

Our segmentation model claims an important role of the curvature variation of the interplate contact geometry along-strike. Another model suggests that giant earthquakes are propagated along dip flat segments of subduction zones^51^. The shear strength over this type of flat subduction segments is more homogeneous and therefore more likely to be exceeded over large areas. We suggest that along-strike straight subduction segments behave in a similar manner. Also, kinematic models of the main shock and the largest aftershock of Pisagua earthquake sequence suggest that an along-dip segmentation occurred during the propagation of both ruptures^45^. This segmentation has been interpreted as a result of a change of dip angle of the subducting slab. Figure 4D shows the curvature variation of the Chilean subduction zone considering trench, 20 km depth contour and 40 km depth contour and the location of the most recent earthquakes, including the M_w_ 8.8 Maule 2010 earthquake and the M_w_ 8.3 2015 Illapel earthquake. These two earthquakes stop their propagation at major changes in strike of the interplate contact; this observation indicates that along-strike changes in curvature of the interplate contact could control rupture propagation due to variation in shear strength and/or variation in locking.

### Concluding Remarks

Why the seismic gap did not rupture totally during the Pisagua earthquake can be explained by the heterogeneous locking, margin curvature and subducting ridges, which determine heterogeneous prestress levels at the interplate and fault segmentation. The area of the Pisagua earthquake most likely represents a region where the level of prestress was higher since the time of the two largest historical earthquakes. This area probably did not completely slip during the events of 1868 and 1877, and a slip deficit has accumulated since those times. The adjacent segments, given by the areas of the historical earthquake in the south of Peru and in the north of Chile, have increased the potential for earthquake nucleation because the slip deficit still remains high. The better earthquake scenario in terms of impact for the coastal cities of Peru and Chile is that these two adjacent segments rupture separately. However, both segments can rupture together if the slip deficit remains high near the trench in the rupture area of the Pisagua earthquake. The seismic moments released by all the processes involved in the Pisagua earthquake were not able to reduce the accumulated seismic moment deficit of the entire seismic gap. Therefore, we agree that this region still has the potential to generate a great earthquake, as has occurred in the past.

## Supplementary information

Earthquake segmentation in northern Chile correlates with curved plate geometry
