# Supplementary material for: Earthquake segmentation in northern Chile correlates with curved plate geometry
Source: Sci Rep. 2019 Mar 13;9:4403. doi: 10.1038/s41598-019-40282-6 (PMC6416342; doi:10.1038/s41598-019-40282-6)
Supplement: Supplementary file 1 — Earthquake segmentation in northern Chile correlates with curved plate geometry [file 41598_2019_40282_MOESM1_ESM.docx]

**SUPPLEMENTARY INFORMATION**

**Earthquake segmentation in northern Chile correlates with curved plate geometry**

Mahesh N. Shrivastava^1, 2*^, Gabriel González^1, 2^, Marcos Moreno^3, 5^, Hugo Soto^3^, Bernd Schurr^3^, Pablo Salazar^1, 2^, Juan Carlos Báez^4^

^1^National Research Center for Integrated Natural Disaster Management, Chile

^2^Universidad Católica del Norte, Antofagasta, Chile

^3^GFZ Helmholtz Centre Potsdam, German Research Centre for Geosciences, Germany

^4^Centro Sismológico Nacional, Universidad de Chile, Chile

^5^ Departamento de Geofísica, Facultad de Ciencias Físicas y Matemáticas, Universidad de Concepción, Chile

*Corresponding author: [mahesh.shrivastava@ucn.cl](mailto:mahesh.shrivastava@ucn.cl)

**Contents of this file**

Text S1 to S2

Figure S1 to S10

Tables S1 to S4

**Introduction**

This supporting material consists of two texts describing the estimation of cumulative seismic moment and the curvature of the along-strike interplate contact. This supporting information also contains nine supporting figures and four supporting tables.

**Supplementary Text S1**

**Estimation of Cumulative M_o_ and Slip**

We assumed that the local magnitudes calculated for the aftershocks correspond to moment magnitudes due to the moderate sizes of the events. The seismic moment and slip were calculated using scaling relations, which relate the seismic moment with rupture lengths and areas. We calculated the rupture areas (A) of aftershocks using the scaling relations^1^. We calculated the slip for individual aftershocks from their moment magnitude using the equation^2^ to relate the moment magnitude (Mw) to seismic moment (M_0_). We assumed a constant shear modulus μ = ρ*v_s_^2^ = 35 GPa, calculated using v_s_ = 3385 m s^−1^ and ρ = 3050 km m^−3^.

**Supplementary Text S2**

**Estimation of the curvature of the interplate contact along-strike**

The formal estimation of the curvature, *k*, is

$k=\frac{dT}{ds}$

where *T* is the unit tangent and *s* is the arc length.

The curvature of the Andean subduction zone has been calculated to reveal how the 20-km and 40-km slab contours and the trench are changing along strike. We have estimated the along-strike curvature individually for the trench, the 20-km slab contour and the 40-km slab contour. We have also estimated the mean curvature of the trench and the slab contours at depths of 20 and 40 km. The curvatures are shown in figure S6.

The figures were prepared using the GMT software package^3^.

**
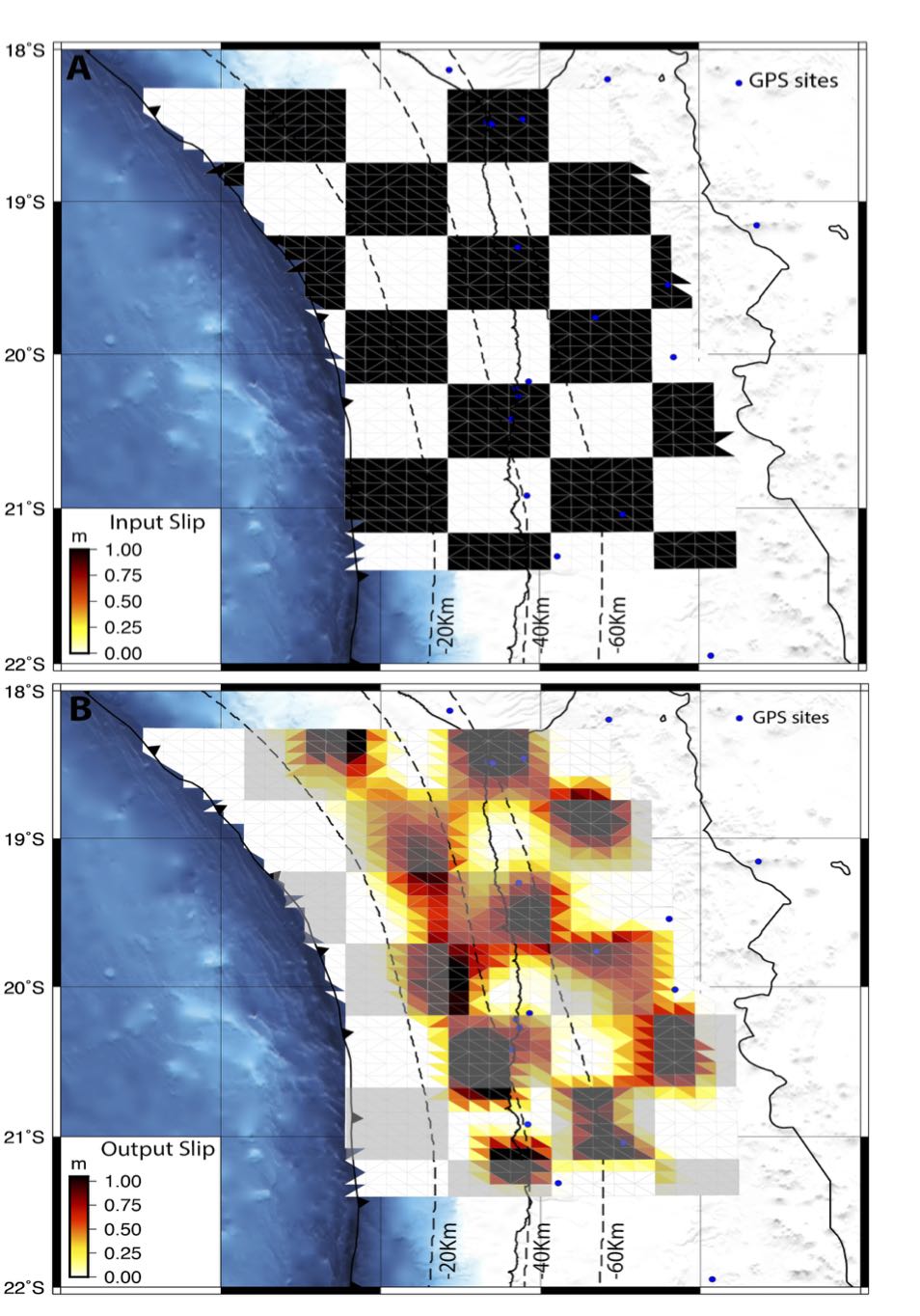
**

**Supplementary Figure S1.** Checkerboard test for the resolution ability of the network and the inversion method to recover the synthetically introduced slip: **A**) initial distribution of checkerboard patches; **B**) result of the preferred model, assuming a smoothing parameter of 𝛾 equal to 17, corresponding to our optimal value. The slab contour line is shown as a black dotted line with a contour spacing of 20 km. The gray shaded patches overlap the recovered slip regions.


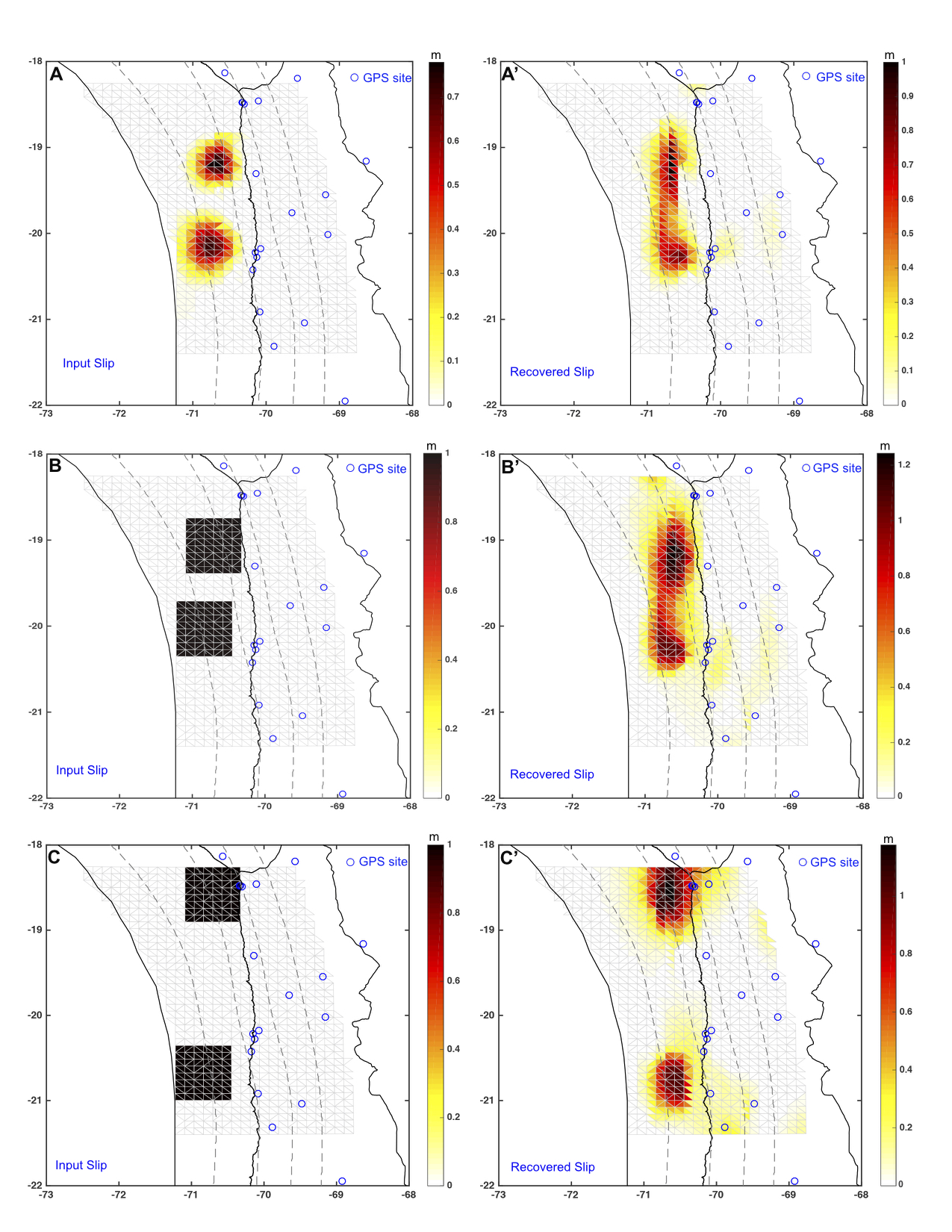


**Supplementary Figure S2.** Checkerboard test for the resolution ability of the network and the inversion method to recover the synthetically introduced slip: **A**) initial distribution of afterslip as the input slip and A’) as the recovered slip; **B**) the input of homogenous patch slip as 1 m in the region of high afterslip and B’) as the recovered slip; **C)** the input of homogenous patch slip shifted north and south region and C’) the recovered slip with the preferred model, assuming a smoothing parameter of 𝛾 equal to 17, corresponding to our optimal value. The slab contour line is shown as a black dotted line with a contour spacing of 20 km. The gray shaded patches overlap the recovered slip regions.


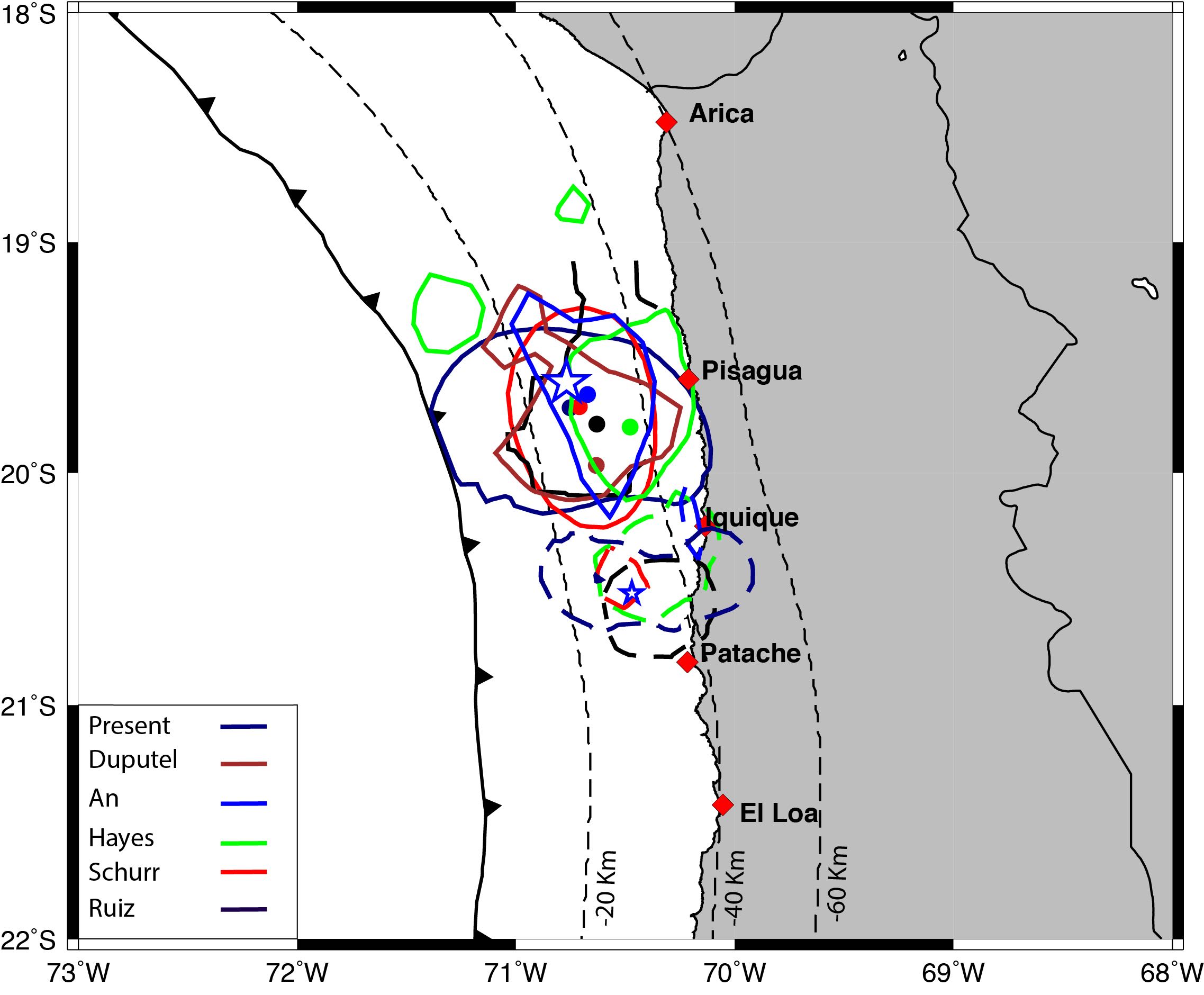


**Supplementary Figure S3.** The coseismic slip distributions compared with published coseismic slip with contour 2 m and the maximum slip regions with closed dots and the largest aftershock slip contour 1 m with dotted line. The contour and closed dotted colored lines correspond to the results from the authors named in the figure.

**
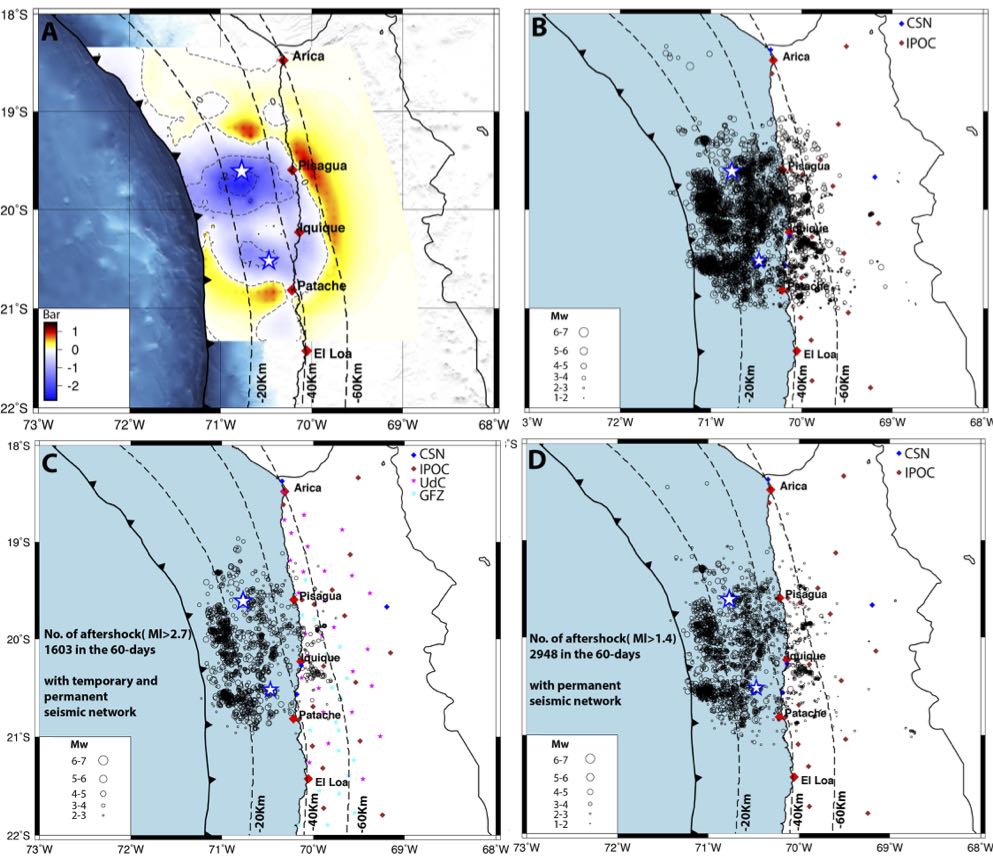
**

**Supplementary Figure S4** (A) Combined Coulomb stress changes estimated from the modeled coseismic slip of the mainshock and the largest aftershock of the 2014 Pisagua earthquake. The blue stars show the epicenter of the 2014 Pisagua earthquake and the largest aftershock. (B) Aftershock locations from the continuous seismic networks from the largest aftershock to the end of the year 2014 from data^4^. The minimum magnitude of aftershock is Ml 1.4 and completeness of seismicity 2.8 (C) The aftershocks locations from the continuous and temporary seismic networks from the largest aftershock at 60 days. The minimum magnitude of aftershock is Ml 2.7. (D) The aftershocks locations 60 days after the largest aftershock, for comparing the continuous and temporary seismic networks. The blue and brown diamonds represent the permanent seismic network, and the cyan and magenta stars represent the temporary seismic network set up after the Pisagua earthquake.

**
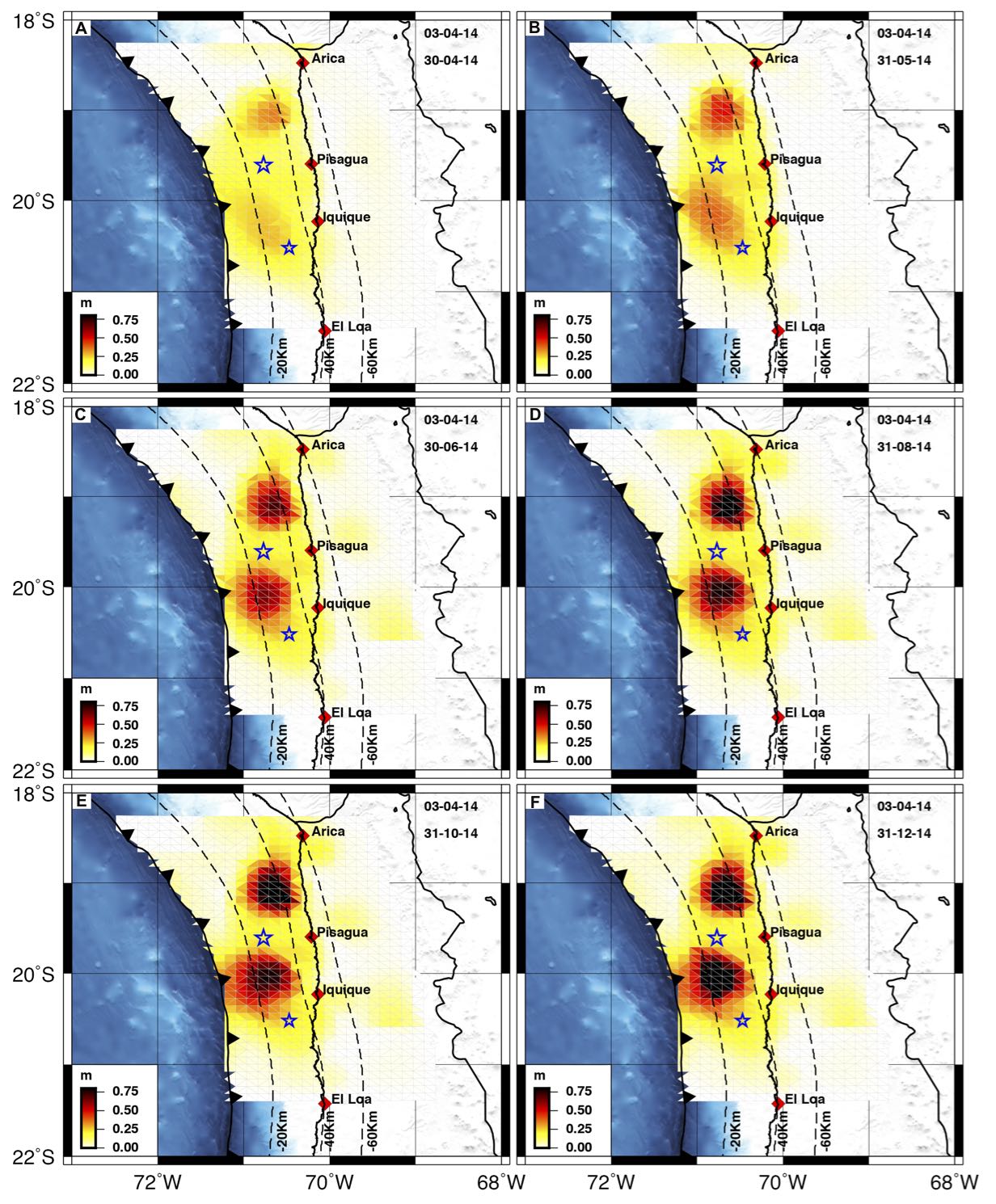
**

**Supplementary Figure S5.** Figures showing the evolution of the afterslip as subplots. First subplot (**A**) evolution of the afterslip in the 28 days after the largest aftershock, from April 3^rd^ to April 30^th^, 2014; subplot **(B**) evolution of the afterslip 59 days after the largest aftershock, from April 3^rd^ to May 31^st^, 2014; (**C**) evolution of the afterslip in the 89 days after the largest aftershock, from April 3^rd^ to June 30^th^, 2014; subplot **(D**) evolution of the afterslip 150 days after the largest aftershock, from April 3^rd^ to August 31^st^, 2014; (**E**) evolution of the afterslip in the 211 days after the largest aftershock, from April 3^rd^ to October 31^st^, 2014; subplot **(F**) evolution of the afterslip 273 days after the largest aftershock, from April 3^rd^ to December 31^st^, 2014.

**
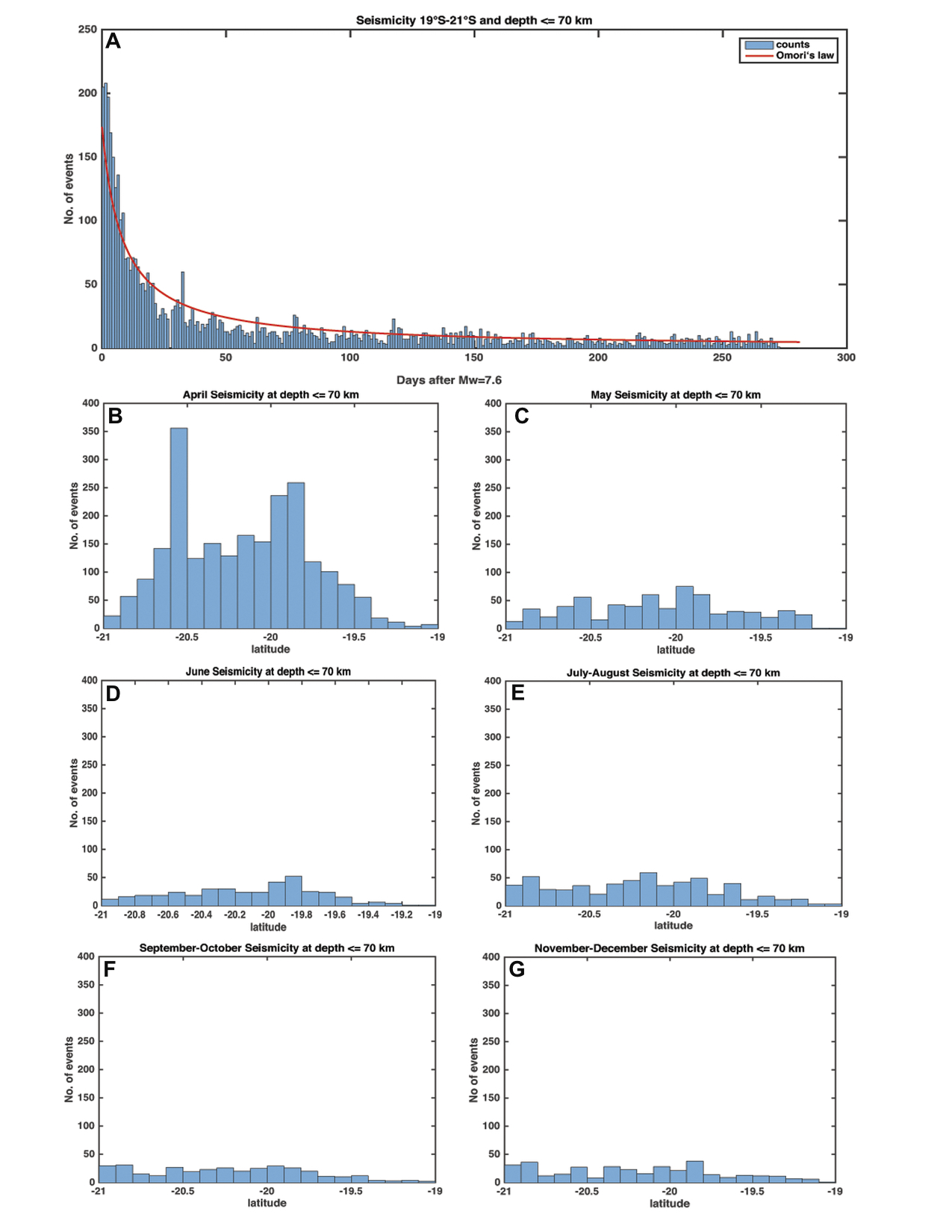
**

**Supplementary Figure S6. A)** The aftershock distribution with time and curve fit with modified Omori’s law. The best fit with modified Omori’s law has decay rate ‘p’ 1. B), C), D), E), F) and G) show the monthly aftershock occurrence.


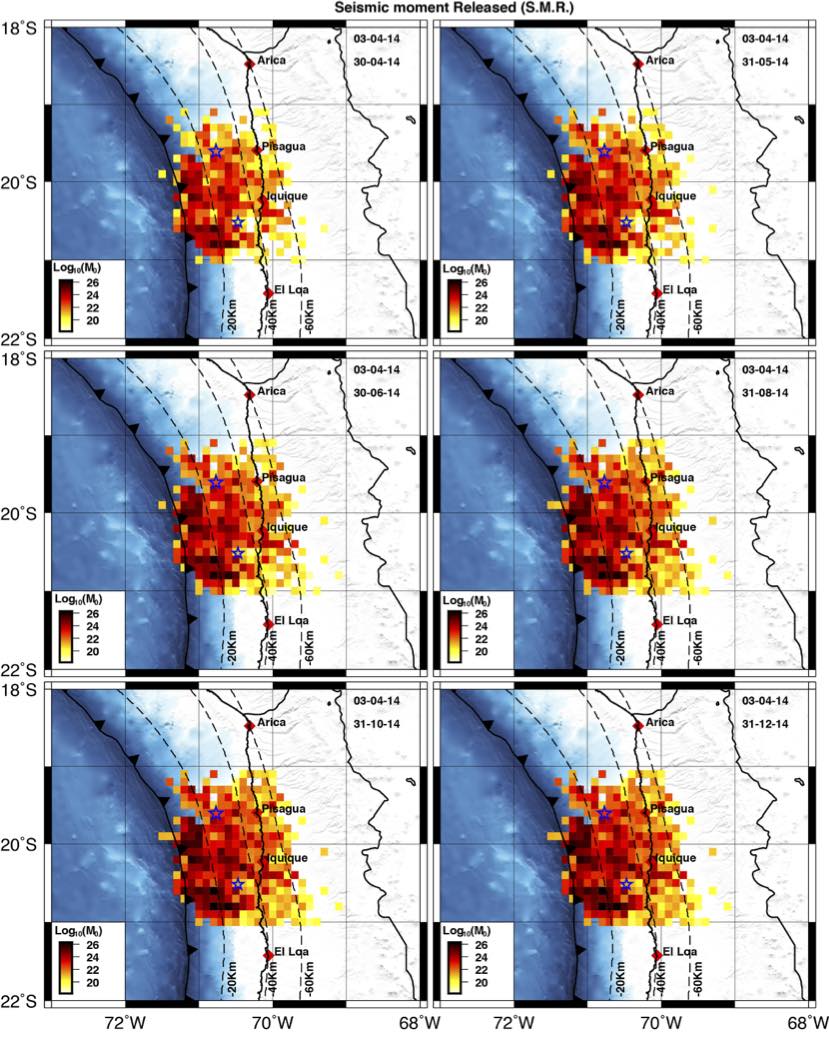


**Supplementary Figure S7.** Figures showing the seismic moment released with aftershocks corresponding to the time frame of Figure S5.

**
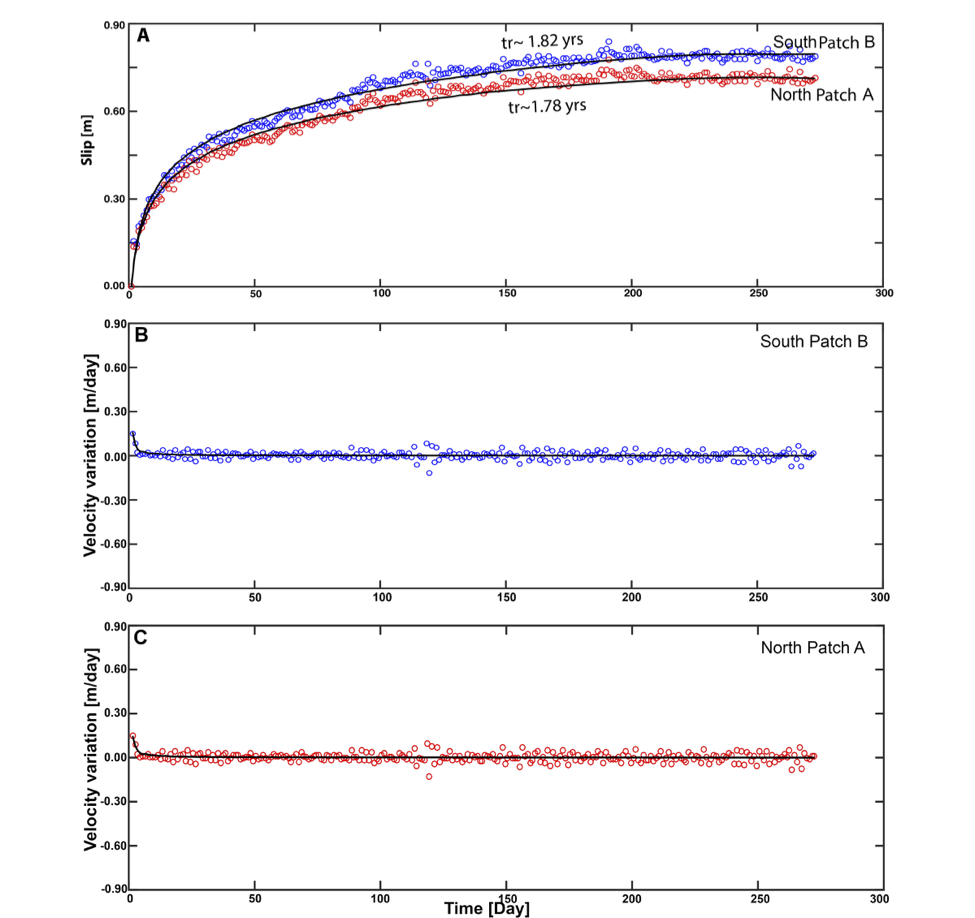
**

**Supplementary Figure S8 A**) The evolution of afterslip at the location of peak afterslip in both patches A and B. This time evolution is well-fit using the analytical law^5^ and provides relaxation times of *t_r_* = 1.82 yr and 1.78 yr for the north and south patches, respectively. **B)** and **C)** show the velocity variations for patches B and A, respectively.

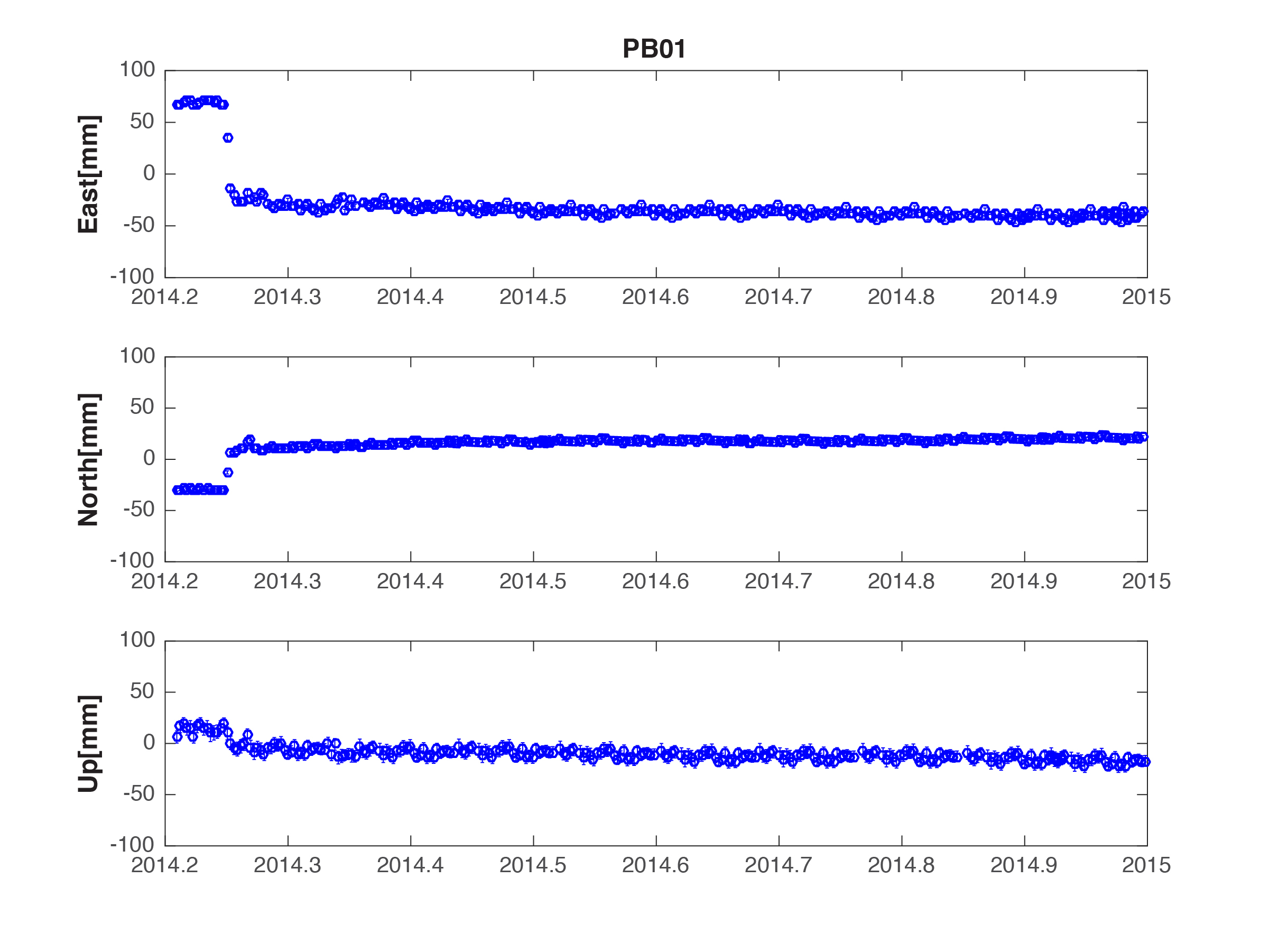


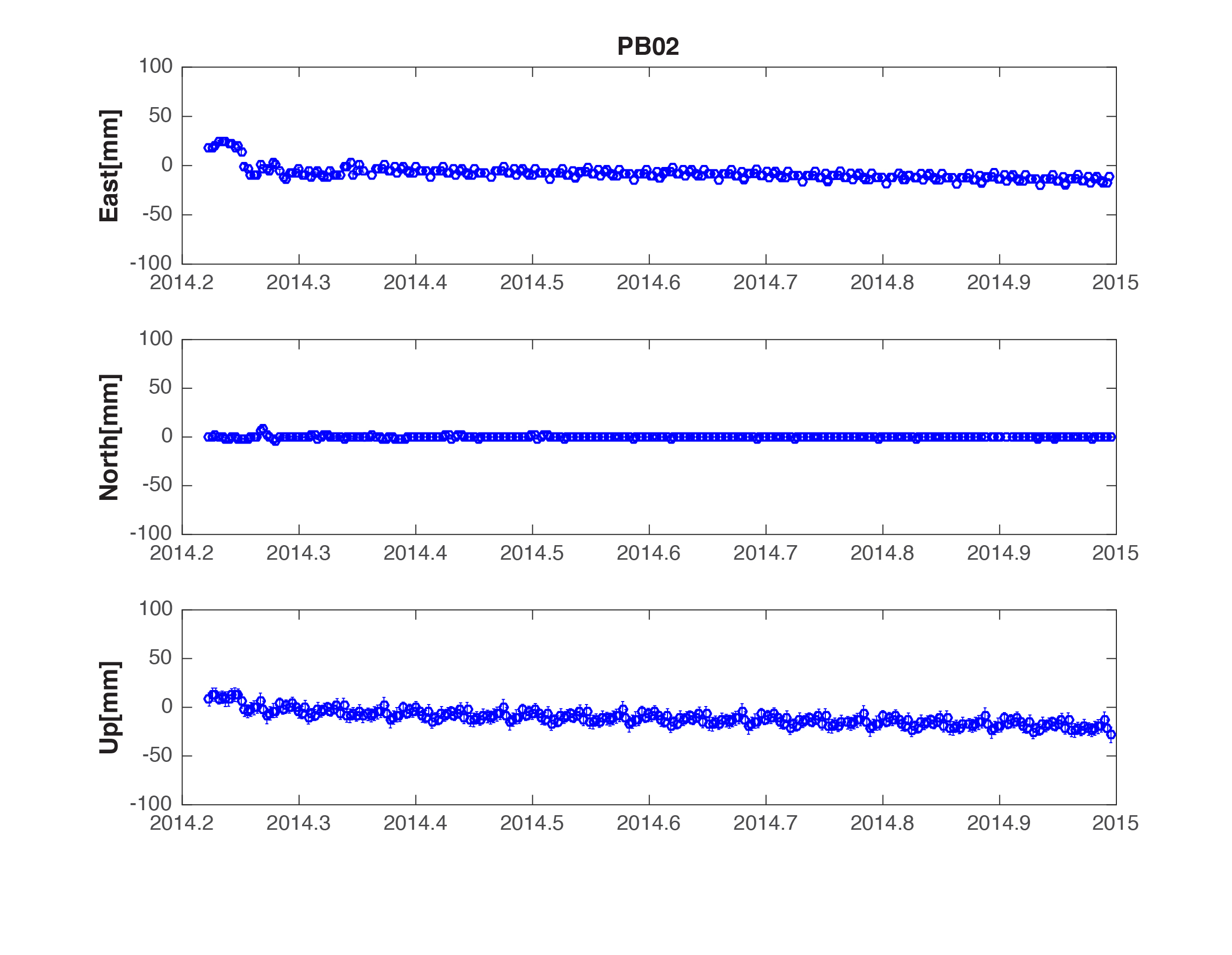


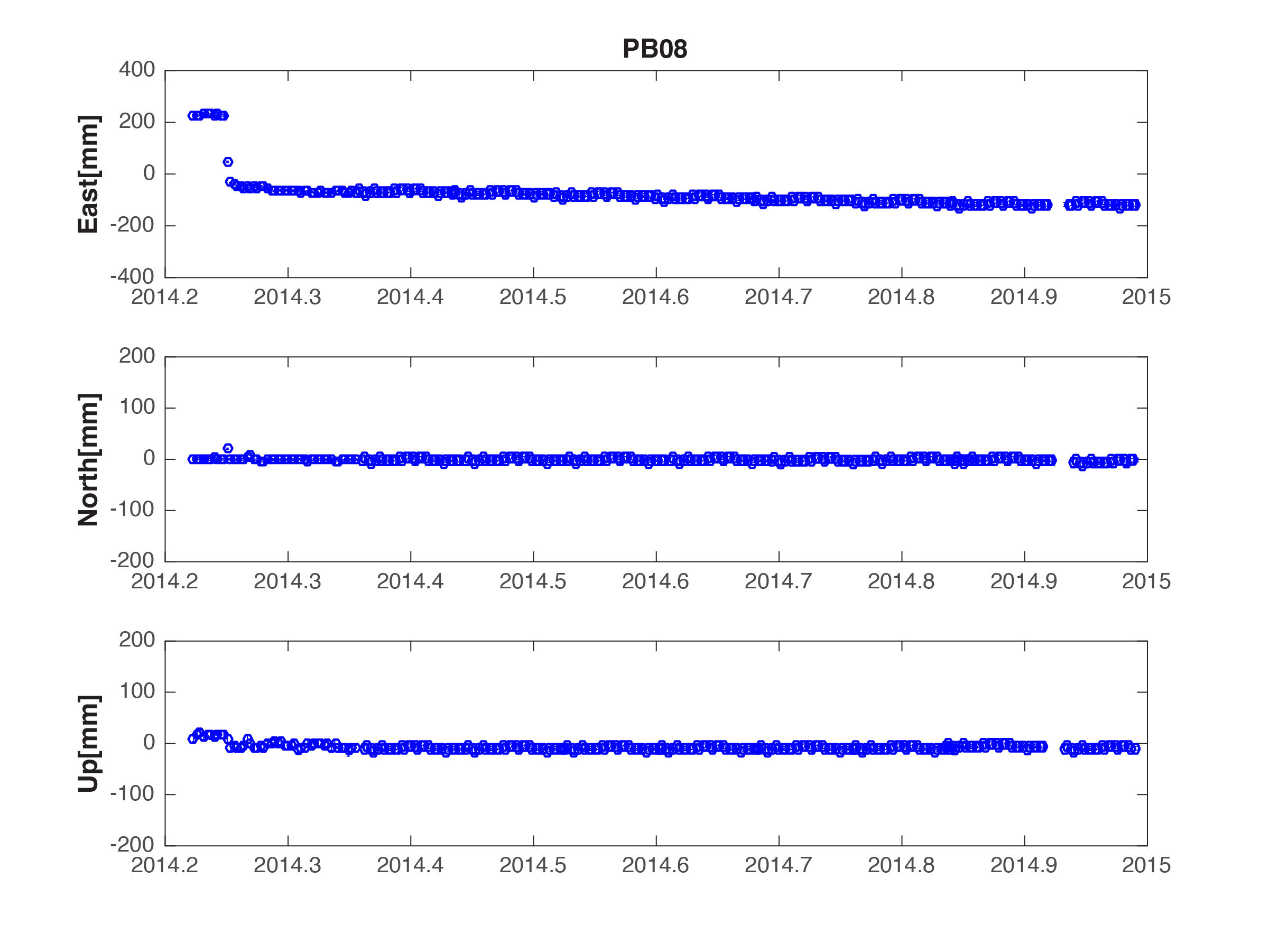


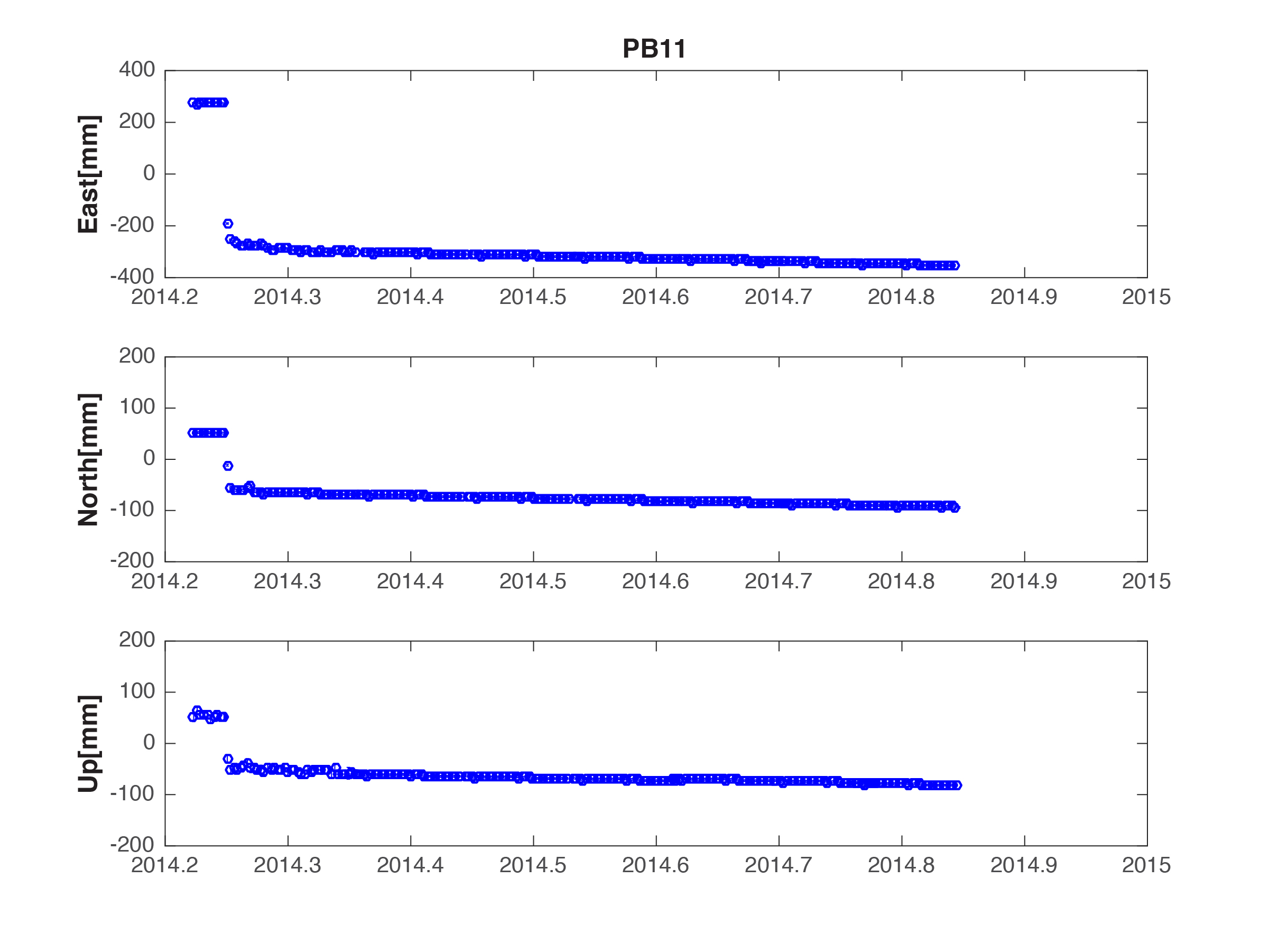

Supp**lementary Figure S9.** Figures showing the GPS time series repeatability with errors, which we used to model the coseismic slip and afterslip distribution. We have detrended the time series and removed the mean value.


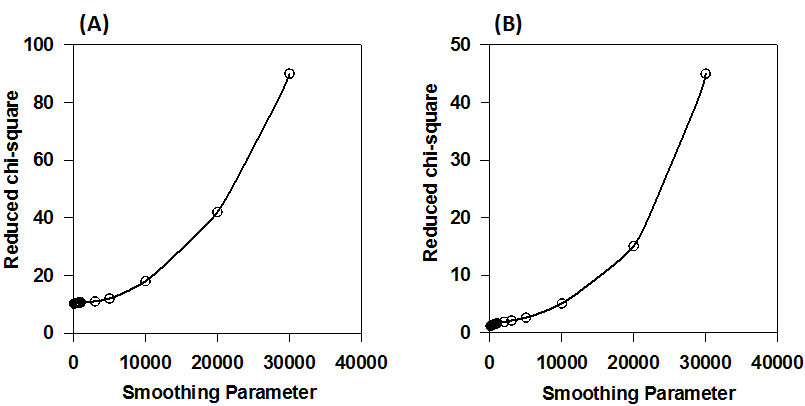


**Supplementary Figure S10.** Models of the coseismic slip of the mainshock and the largest aftershock, assuming a smoothing parameter of 𝛾 equal to 17, corresponding to our optimal value. For models of afterslip, we assumed a smoothing parameter of 𝛾 equal to 1.6, corresponding to our optimal value.

**Supplementary Table S1.** The observed and modeled coseismic displacements of the mainshock of the 2014 Pisagua earthquake.

| **S/N.** | **GPS**  **Sites** | **Lon**  **(deg)** | **Lat**  **(deg)** | **Observed displacement (m)** | | | **Model displacement (m)** | | |
| --- | --- | --- | --- | --- | --- | --- | --- | --- | --- |
|  |  |  |  | **East** | **North** | **Up** | **East** | **North** | **Up** |
| 1 | AEDA | -70.17 | -20.42 | -0.2022 | 0.0510 | -0.0601 | -0.2212 | 0.0576 | -0.0551 |
| 2 | ATJN | -70.13 | -19.30 | -0.5484 | -0.2407 | -0.1551 | -0.5419 | -0.2474 | -0.1743 |
| 3 | BN01 | -70.15 | -20.21 | -0.3571 | 0.0344 | -0.0459 | -0.3559 | 0.0529 | -0.0703 |
| 4 | BN15 | -70.32 | -18.47 | -0.0526 | -0.0682 | -0.0232 | -0.0572 | -0.0527 | -0.0201 |
| 5 | CGTC | -70.06 | -20.17 | -0.4073 | 0.0677 | -0.0850 | -0.3849 | 0.0600 | -0.0697 |
| 6 | CHM2 | -69.19 | -19.54 | -0.2299 | -0.0339 | -0.0105 | -0.2236 | -0.0347 | -0.0398 |
| 7 | COL1 | -68.63 | -19.15 | -0.1040 | -0.0303 | 0.0044 | -0.1105 | -0.0304 | -0.0080 |
| 8 | CRSC | -70.08 | -20.91 | -0.0158 | -0.0010 | -0.0108 | -0.0340 | 0.0045 | -0.0180 |
| 9 | IAC1 | -70.33 | -18.48 | -0.0510 | -0.0653 | -0.0329 | -0.0565 | -0.0516 | -0.0200 |
| 10 | IQQE | -70.13 | -20.27 | -0.2843 | 0.0346 | -0.0497 | -0.3081 | 0.0484 | -0.0625 |
| 11 | LYAR | -70.56 | -18.13 | -0.0104 | -0.0156 | -0.0199 | -0.0186 | -0.0112 | -0.0147 |
| 12 | PB01 | -69.48 | -21.04 | -0.0328 | 0.0173 | -0.0085 | -0.0485 | 0.0231 | -0.0025 |
| 13 | PB02 | -69.89 | -21.31 | -0.0076 | 0.0000 | -0.0062 | -0.0186 | 0.0020 | -0.0094 |
| 14 | PB08 | -69.16 | -20.01 | -0.1787 | 0.0201 | -0.0088 | -0.1950 | 0.0163 | -0.0388 |
| 15 | PB11 | -69.65 | -19.76 | -0.3664 | -0.0092 | -0.0825 | -0.3807 | -0.0108 | -0.0460 |
| 16 | PCCL | -70.10 | -18.45 | -0.0680 | -0.0734 | -0.0135 | -0.0686 | -0.0673 | -0.0211 |
| 17 | PTRE | -69.57 | -18.19 | -0.0460 | -0.0529 | 0.0022 | -0.0577 | -0.0511 | -0.0118 |
| 18 | RADO | -68.92 | -21.94 | 0.0026 | 0.0017 | 0.0008 | -0.0117 | 0.0030 | -0.0034 |
| 19 | UTAR | -70.29 | -18.49 | -0.0564 | -0.0695 | -0.0283 | -0.0607 | -0.0565 | -0.0205 |

**Supplementary Table S2.** The observed and modeled coseismic displacements of the largest aftershock of the 2014 Pisagua earthquake.

| **S/N.** | **GPS Sites** | Lon  (deg) | Lat  (deg) | **Observed displacement (m)** | | | **Model displacement (m)** | | |
| --- | --- | --- | --- | --- | --- | --- | --- | --- | --- |
|  |  |  |  | East | North | Up | East | North | Up |
| 1 | AEDA | -70.17 | -20.42 | -0.2125 | -0.0357 | 0.1006 | -0.2182 | -0.0320 | 0.0730 |
| 2 | ATJN | -70.13 | -19.30 | -0.0188 | -0.0110 | -0.0162 | -0.0194 | -0.0097 | -0.0105 |
| 3 | BN01 | -70.15 | -20.21 | -0.1794 | -0.0280 | 0.0385 | -0.1767 | -0.0294 | 0.0376 |
| 4 | BN15 | -70.32 | -18.47 | -0.0049 | -0.0029 | -0.0070 | -0.0049 | -0.0032 | -0.0045 |
| 5 | CGTC | -70.06 | -20.17 | -0.1461 | -0.0374 | -0.0088 | -0.1518 | -0.0362 | 0.0049 |
| 6 | CHM2 | -69.19 | -19.54 | -0.0351 | -0.0147 | -0.0011 | -0.0402 | -0.0249 | -0.0094 |
| 7 | COL1 | -68.63 | -19.15 | -0.0060 | 0.0003 | 0.0048 | -0.0196 | -0.0116 | -0.0020 |
| 8 | CRSC | -70.08 | -20.91 | -0.0635 | -0.0216 | -0.0140 | -0.0650 | -0.0194 | 0.0021 |
| 9 | IAC1 | -70.33 | -18.48 | -0.0044 | -0.0038 | -0.0022 | -0.0049 | -0.0031 | -0.0045 |
| 10 | IQQE | -70.13 | -20.27 | -0.1944 | -0.0311 | 0.0475 | -0.1885 | -0.0313 | 0.0381 |
| 11 | LYAR | -70.56 | -18.13 | 0.0002 | -0.0039 | -0.0043 | -0.0015 | -0.0003 | -0.0027 |
| 12 | PB01 | -69.48 | -21.04 | -0.0487 | 0.0184 | -0.0091 | -0.0487 | 0.0178 | -0.0167 |
| 13 | PB02 | -69.89 | -21.31 | -0.0143 | -0.0002 | -0.0089 | -0.0222 | 0.0033 | -0.0108 |
| 14 | PB08 | -69.16 | -20.01 | -0.0813 | -0.0199 | -0.0165 | -0.0732 | -0.0246 | -0.0203 |
| 15 | PB11 | -69.65 | -19.76 | -0.0624 | -0.0425 | -0.0214 | -0.0631 | -0.0431 | -0.0248 |
| 16 | PCCL | -70.10 | -18.45 | -0.0033 | -0.0074 | -0.0112 | -0.0056 | -0.0046 | -0.0039 |
| 17 | PTRE | -69.57 | -18.19 | -0.0046 | -0.0062 | -0.0117 | -0.0053 | -0.0053 | -0.0020 |
| 18 | RADO | -68.92 | -21.94 | 0.0092 | 0.0149 | 0.0064 | -0.0080 | 0.0042 | -0.0027 |
| 19 | UTAR | -70.29 | -18.49 | -0.0050 | -0.0043 | -0.0083 | -0.0052 | -0.0035 | -0.0045 |

**Supplementary Table S3.** The observed and modeled cumulative displacements for 273 days after the largest aftershock.

| **No.** | **GPS Sites** | Lon  (deg) | Lat  (deg) | **Observed displacement (m)** | | | **Model displacement (m)** | | |
| --- | --- | --- | --- | --- | --- | --- | --- | --- | --- |
|  |  |  |  | East | North | Up | East | North | UP |
| 1 | AEDA | -70.17 | -20.42 | -0.0871 | -0.0029 | -0.0330 | -0.0871 | -0.0029 | -0.0330 |
| 2 | ATJN | -70.13 | -19.30 | -0.0872 | -0.0184 | -0.0323 | -0.0865 | -0.0222 | -0.0320 |
| 3 | BN01 | -70.15 | -20.21 | -0.0927 | -0.0111 | -0.0202 | -0.0915 | -0.0123 | -0.0277 |
| 4 | BN15 | -70.32 | -18.47 | -0.0279 | -0.0182 | -0.0029 | -0.0265 | -0.0172 | -0.0060 |
| 5 | CGTC | -70.06 | -20.17 | -0.1023 | -0.0082 | -0.0215 | -0.1007 | -0.0097 | -0.0177 |
| 6 | CHM2 | -69.19 | -19.54 | -0.0309 | -0.0061 | -0.0068 | -0.0309 | -0.0061 | -0.0061 |
| 7 | COL1 | -68.63 | -19.15 | -0.0096 | -0.0035 | -0.0001 | -0.0070 | -0.0025 | -0.0060 |
| 8 | CRSC | -70.08 | -20.91 | -0.0327 | 0.0017 | -0.0044 | -0.0314 | 0.0014 | -0.0065 |
| 9 | IAC1 | -70.33 | -18.48 | -0.0260 | -0.0169 | -0.0066 | -0.0269 | -0.0175 | -0.0049 |
| 10 | IQQE | -70.13 | -20.27 | -0.0919 | -0.0101 | -0.0137 | -0.0966 | -0.0113 | -0.0215 |
| 11 | LYAR | -70.56 | -18.13 | -0.0091 | -0.0039 | -0.0020 | -0.0114 | -0.0034 | -0.0001 |
| 12 | PB01 | -69.48 | -21.04 | -0.0141 | 0.0064 | -0.0043 | -0.0169 | 0.0051 | -0.0013 |
| 13 | PB02 | -69.89 | -21.31 | -0.0112 | 0.0039 | -0.0082 | -0.0112 | 0.0039 | -0.0080 |
| 14 | PB08 | -69.16 | -20.01 | -0.0275 | -0.0008 | -0.0083 | -0.0280 | -0.0047 | -0.0026 |
| 15 | PB11 | -69.65 | -19.76 | -0.0342 | -0.0106 | -0.0123 | -0.0340 | -0.0114 | -0.0135 |
| 16 | PCCL | -70.10 | -18.45 | -0.0375 | -0.0215 | -0.0066 | -0.0367 | -0.0207 | -0.0144 |
| 17 | PTRE | -69.57 | -18.19 | -0.0241 | -0.0203 | -0.0109 | -0.0280 | -0.0189 | -0.0097 |
| 18 | RADO | -68.92 | -21.94 | -0.0085 | 0.0015 | -0.0012 | -0.0078 | 0.0015 | -0.0010 |
| 19 | UTAR | -70.29 | -18.49 | -0.0343 | -0.0306 | 0.0082 | -0.0348 | -0.0293 | -0.0074 |

**Supplementary Table S4.** The seismic networks, IPOC and CSN seismic sites are permanent, whereas UdC and GFZ were installed after the Pisagua earthquake.

| S/N | Seismic  Sites | Location | | Alt.  (m) | S/N | Seismic  Sites | Location | | Alt.  (m) |
| --- | --- | --- | --- | --- | --- | --- | --- | --- | --- |
|  |  | Lon(d) | Lat(d) |  |  |  | Lon(d) | Lat(d) |  |
| **UdC** | | | | |  | | | | |
| 1. | IN01 | -70.04 | -20.17 | 726 | 42. | LT13 | -69.72 | -21.14 | 858 |
| 2. | IN02 | -69.89 | -20.74 | 989 | 43. | LT14 | -69.93 | -21.55 | 955 |
| 3. | IN03 | -70.08 | -19.94 | 680 | 44. | LT15 | -69.71 | -21.58 | 1231 |
| 4. | IN04 | -69.57 | -19.34 | 2201 | 45. | LT16 | -69.54 | -21.77 | 1020 |
| 5. | IN05 | -69.40 | -20.10 | 1713 | 46. | LT17 | -69.84 | -21.89 | 1790 |
| 6. | IN06 | -69.87 | -19.61 | 1194 | 47. | LT18 | -70.10 | -22.05 | 1240 |
| 7. | IN07 | -69.77 | -19.87 | 1161 | 48. | LT19 | -70.16 | -21.89 | 142 |
| 8. | IN08 | -69.56 | -20.33 | 1018 | 49. | LT20 | -69.83 | -22.24 | 1600 |
| 9. | IN09 | -70.25 | -19.18 | 58 | 50. | LT21 | -69.49 | -22.12 | 1120 |
| 10. | IN10 | -69.87 | -19.29 | 1311 | 51. | LT22 | -69.47 | -22.45 | 1295 |
| 11. | IN11 | -69.83 | -21.06 | 934 | 52. | LT23 | -69.87 | -22.86 | 1580 |
| 12. | IN12 | -69.37 | -19.52 | 2721 | 53. | LT24 | -69.75 | -20.85 | 1121 |
| 13. | IN13 | -70.12 | -20.39 | 877 | **IPOC** | | | | |
| 14. | IN14 | -69.57 | -20.74 | 976 | 54. | CORZ | -69.79 | -19.49 | 0 |
| 15. | IN15 | -69.45 | -21.43 | 862 | 55. | PATA | -70.00 | -20.69 | 780 |
| 16. | IN16 | -69.93 | -20.50 | 1044 | 56. | CHOM | -70.01 | -21.09 | 1200 |
| 17. | IN17 | -69.35 | -20.48 | 1278 | 57. | ANIT | -69.99 | -19.64 | 969 |
| 18. | IN18 | -69.26 | -20.99 | 1444 | 58. | CANO | -69.53 | -20.44 | 994 |
| 19. | IN19 | -70.23 | -18.90 | 879 | 59. | PB01 | -69.48 | -21.04 | 900 |
| 20. | IN20 | -70.10 | -18.72 | 987 | 60. | PB02 | -69.89 | -21.31 | 1015 |
| 21. | IN21 | -70.32 | -18.48 | 127 | 61. | PB03 | -69.75 | -22.04 | 1460 |
| 22. | IN22 | -70.31 | -18.77 | 128 | 62. | PB04 | -70.14 | -22.33 | 1520 |
| 23. | IN23 | -69.68 | -18.87 | 2402 | 63. | PB05 | -70.20 | -22.85 | 1150 |
| 24. | IN24 | -70.04 | -21.26 | 998 | 64. | PB06 | -69.57 | -22.70 | 1440 |
| 25. | IN25 | -69.90 | -19.95 | 1125 | 65. | PB07 | -69.88 | -21.72 | 1570 |
| 26. | IN26 | -70.06 | -19.04 | 1172 | 66. | PB08 | -69.15 | -20.14 | 3060 |
| 27. | IN27 | -70.15 | -19.52 | 964 | 67. | PB09 | -69.24 | -21.79 | 1530 |
| 28. | IN28 | -70.10 | -19.30 | 1328 | 68. | PB10 | -70.55 | -23.51 | 250 |
| 29. | IN33 | -70.10 | -18.72 | 987 | 69. | PB11 | -69.65 | -19.76 | 1360 |
| **GFZ** | | | | | 70. | PB12 | -70.32 | -18.61 | 908 |
| 30. | LT01 | -69.61 | -21.39 | 776 | 71. | PB14 | -70.40 | -24.62 | 2600 |
| 31. | LT02 | -69.55 | -21.23 | 794 | 72. | PB15 | -69.47 | -23.20 | 1830 |
| 32. | LT03 | -69.63 | -20.40 | 990 | 73. | PB16 | -69.50 | -18.33 | 4480 |
| 33. | LT04 | -69.70 | -20.59 | 901 | 74. | PATCX | -70.15 | -20.82 | 830 |
| 34. | LT05 | -69.79 | -20.49 | 1015 | 75. | HMBCX | -69.88 | -20.27 | 1152 |
| 35. | LT06 | -69.70 | -20.07 | 1099 | 76. | MNMCX | -69.59 | -19.13 | 2304 |
| 36. | LT07 | -69.99 | -19.79 | 1376 | 77. | PSGCX | -70.12 | -19.59 | 966 |
| 37. | LT08 | -70.11 | -19.75 | 1034 |  | **CSN** | | | |
| 38. | LT09 | -70.08 | -19.39 | 1155 | 78. | AP01 | -70.34 | -18.37 | 31 |
| 39. | LT10 | -69.85 | -20.15 | 1143 | 79. | TA01 | -70.13 | -20.27 | 86 |
| 40. | LT11 | -69.72 | -20.93 | 980 | 81. | TA02 | -70.18 | -20.56 | 616 |
| 41. | LT12 | -70.03 | -20.93 | 680 | 82. | GO01 | -69.19 | -19.66 | 3809 |

**References:**

1. Wells, D. L., & Coppersmith, K. J. New empirical relationships among magnitude, rupture length, rupture width, rupture area, and surface displacement. *Bull. Seismo. Soc. Am.* *84*(4), 974-1002(1994).

2. Kanamori, H. The energy release in great earthquakes. *J. Geophys. Res.*, 82(20), 2981-2987(1997).

3. Wessel, P., & Smith, W. H. The Generic Mapping Tools*, GMT, Version 4.5. 15: Technical Reference and Cookbook*. School of Ocean and Earth Science and Technology, University of Hawaii at Manoa, (2016).

4. Sippl, C., Schurr, B., Asch, G., & Kummerow, J. Seismicity structure of the Northern Chile forearc from>100,000 double‐difference relocated hypocenters. *J.Geophys. Res.*,123, 4063–4087(2018).

5. Perfettini, H., & Avouac, J. P. Postseismic relaxation driven by brittle creep: A possible mechanism to reconcile geodetic measurements and the decay rate of aftershocks, application to the Chi-Chi earthquake, Taiwan, *J. Geophys. Res.* 109(B2) (2004).
